# Perceptual category learning of photographic and painterly stimuli in rhesus macaques (*Macaca mulatta*) and humans

**DOI:** 10.1371/journal.pone.0185576

**Published:** 2017-09-29

**Authors:** Drew Altschul, Greg Jensen, Herbert Terrace

**Affiliations:** 1 The University of Edinburgh, Department of Psychology, 7 George Square, Edinburgh, United Kingdom; 2 Scottish Primate Research Group, Scotland, United Kingdom; 3 Centre for Cognitive Ageing and Cognitive Epidemiology, 7 George Square, Edinburgh, United Kingdom; 4 Columbia University, Department of Psychology, New York, NY, United States of America; Centre de neuroscience cognitive, FRANCE

## Abstract

Humans are highly adept at categorizing visual stimuli, but studies of human categorization are typically validated by verbal reports. This makes it difficult to perform comparative studies of categorization using non-human animals. Interpretation of comparative studies is further complicated by the possibility that animal performance may merely reflect reinforcement learning, whereby discrete features act as discriminative cues for categorization. To assess and compare how humans and monkeys classified visual stimuli, we trained 7 rhesus macaques and 41 human volunteers to respond, in a specific order, to four simultaneously presented stimuli at a time, each belonging to a different perceptual category. These exemplars were drawn at random from large banks of images, such that the stimuli presented changed on every trial. Subjects nevertheless identified and ordered these changing stimuli correctly. Three monkeys learned to order naturalistic photographs; four others, close-up sections of paintings with distinctive styles. Humans learned to order both types of stimuli. All subjects classified stimuli at levels substantially greater than that predicted by chance or by feature-driven learning alone, even when stimuli changed on every trial. However, humans more closely resembled monkeys when classifying the more abstract painting stimuli than the photographic stimuli. This points to a common classification strategy in both species, one that humans can rely on in the absence of linguistic labels for categories.

## Introduction

When a human perceives a stimulus, it is likely that she will automatically identify that stimulus as belonging to one or more categories. We can describe this process linguistically: when we perceive something, we do not simply see the thing; we see it as a type of thing, part of a set of things which can in some way be treated as equivalent [1]. Categorization of visual stimuli occurs so rapidly in humans that it occurs almost instantaneously [2], and we interpret the world through a diverse set of classifications [3].

Do non-human animals (hereafter, “animals”) possess the same categorization abilities as humans? This question has motivated considerable research by comparative psychologists, who have sought to understand the evolutionary roots of categorization through the study of many species. The resulting literature has demonstrated that animals have the ability to categorize a bewildering range of stimuli, including organic forms, such as faces [4], plants, and animals [5–7], as well as man-made objects, such as cars, chairs [8], orthographic characters [9], paintings [10], cartoons [11], and abstract forms [12]. Animals can also correctly categorize never-before-seen exemplars, showing that this ability is not limited to previously-learned stimuli [13–14]. Perhaps most impressively, animals can categorize images based only on exposures lasting less than 100ms [15], suggesting that animals can process some image features in parallel, rather than performing a systematic visual search for feature cues. These sophisticated abilities have been reviewed extensively elsewhere [16–19].

Researchers of human cognition distinguish categories from concepts: a category is most often defined as a set of entities which exists in the real world and are grouped together, whereas a concept has a more abstract character. It is generally agreed that a “concept” encodes the mental representation used in human categorization [3]. Researchers take for granted that humans possess countless internal concepts, but no such consensus exists for whether animals make use of similarly abstract frameworks. Thus, although animals can clearly categorize stimuli, studying the underlying mechanisms is complicated by the difficulty in disentangling the cognitive representations employed by animal minds from other unrelated features of behavior.

Some authors have defined concepts as necessarily linguistic *a priori*, ruling out mechanisms for non-linguistic concept formation [20], and thereby, all potential models for animal concepts. Other authors have argued that animals learn to categorize using reinforcement learning and associative conditioning alone [21]. This associative account of an animal’s classification of similar stimuli (as described by Herrnstein et al. [22]) relies on the associative strength of discriminable features common across a category [23]. In the absence of verbal communication with their subjects, comparative psychologists have largely limited their inquiries to studies of rule-based concepts [24–25]. While an animal can demonstrate that it has learned the rules imposed by researchers, it hasn’t proved possible to study unsupervised and unrewarded assignment of stimuli into categorical groupings without using language to instruct subjects.

The non-human aptitude for categorization thus falls into a middle ground between associative learning at one extreme and linguistic abstraction at another. Many studies argue that more flexible and sophisticated processes than associative learning are required to explain performance [19]. However, non-human subjects do not possess language, and thus cannot rely on it as a scaffold for categorical inference. The middle ground between these extremes is not well-defined, and no consensus has emerged regarding the appropriate terminology for identifying or describing these cognitive processes.

Herrnstein [26] attempted to build a bridge between the linguistic and associative accounts by proposing that animals classify stimuli using *open-ended categories*. These stimulus clusters would hypothetically rely on the similarities between many learned exemplars, but would fall short of fully-fledged concepts. The criteria for this distinction are vague: a discrimination could be attributed to a concept only if a characteristic other than similarity was used to classify novel exemplars. Skeptics retorted that, because stimuli must necessarily have *some* features in common (without which they would be unrecognizable), those features must permit categorization on the basis of their similarity [27]. Thus, Herrnstein’s proposal left the issue unresolved, and the possibility of feature-driven learning remains a major confound for the study of concept formation in animals.

Herrnstein’s terminology has defined the debate on animal categorization and concept formation, but these are at odds with the most common definitions used among researchers of human cognition. For comparative psychologists, possessing an internal representation that allows an animal to reliably classify stimuli does not immediately indicate that the animal has formed a concept. It is intuitive and well documented that adult humans can flexibly and abstractly manipulate their internal concepts [28], and can use these concepts to mediate their perceptual categorization [24]. The majority of studies of adult human cognition rely on language, but without either language or the ability to directly inquire into the nature of an animal’s mental representation, inference is restricted. Additionally, the explanatory power of associative learning in animals is considered to be very strong by many in the literature [29]. Due to the absence of language and the strong possibility of associative learning, comparative psychologists demand a high level of evidentiary rigor to conclude that animals can form a concept.

By contrast, it is uncontroversial to assert that animals possess *percepts*, here defined as integrated sensory representations constructed from both primary sensory data and top-down information processing [30–32]. Because percepts involve the hierarchical integration of information from different levels of processing, they constitute a form of representation that is implicit and statistical instead of being explicit and propositional, even in humans [33]. Both humans and rhesus macaques are adept at learning to categorize percepts by integrating information, even in cases where discrete features are not available [34]. Macaques can also assign percepts to appropriate experimenter-defined categories [35]. An important property of percepts is that they are more than a list of isolated features. Sensory integration allows organisms to evaluate percepts as gestalt-like wholes.

We are therefore interested in the question of how subjects judge the percept of a stimulus to belong to a particular category. Borrowing a term from the computer vision literature, any algorithm responsible for categorizing a percept is called a *classifier* [36–37]. The definition is functional, in that any algorithm used for categorization may be called a classifier. Ordinarily, the term is used to denote the broad family of strategies used by computer models with varying degrees of success. The methods used by brains to process gestalt sensory information remain an open research topic [38], but these biological processes may also be labeled as classifiers. A rigorous understanding of a subject’s classifier is equivalent to a robust theory of the cognitive processing that underlies categorization. Thus, another way to frame our research question is to ask, “How do the classifiers used by organisms work, and how do those used by monkeys relate to those used by humans?”

The challenge in answering this question empirically is that there are a variety of classifier to choose from. Many of the tasks used to study classification in animals could potentially be solved in several ways. For example, “binary categorization” tasks (e.g. [13, 39]) present subjects with stimuli that belong to one of two categories (e.g. “Is it a house or a face?”). These tasks can be “solved” if some cue exists that identifies one of the categories. Should such a cue exist (e.g. the presence of shapes with 90-degree angles), a simple associative classifier would be sufficient to choose correctly. In order to rule out trivial classification strategies, a study must have a sufficient level of *task complexity* [40]. Requiring that a subject chose between more than two simultaneously presented categories is one way to increase task complexity.

An improvement on this approach is a “pairwise categorization” task. In this scenario, subjects are trained to classify images into more than two categories, but are ultimately tested on pairs of stimuli. For example, Wasserman et al. [29] trained pigeons to classify images according to different 16 categories, a substantial improvement over binary categorization tasks. However, subjects were still only *tested* on two categories at a time, such that a subject that responded at random would still make correct responses 50% of the time. Pairwise categorizations can be accomplished by a low-reliability classifier, even when over a hundred categories are trained simultaneously (as demonstrated in simulation by Jensen & Altschul [40]). Consequently, *training* many categories is an improvement, but pairwise categorization remains a relatively uninformative test of the resulting classifier.

The strongest test of a subject’s classifier is “simultaneous classification,” which requires that subjects classify *all* stimuli, not just two at a time. To date, only one study reports such a design: Bhatt and colleagues [8] trained pigeons to discriminate between four varieties of stimulus (e.g. photographs of cats, flowers, cars, and chairs). Because these four categories were trained and tested in parallel, simple binary discriminations would be insufficient to explain performance. With a chance error rate of 25% rather than 50%, high-level performance could more easily be attributed to a robust classifier.

Nevertheless, while this study raised the difficulty of stimulus categorization considerably, the issue remains that an associative account could still be built based on discrete features. If, for example, subjects relied on an “eye detector,” that would allow them to distinguish most cats from the remaining three categories, even if no other features of cats were considered. When all stimuli in a category share a common set of discrete features, these features could potentially be associated with reward, without a need for more complex cognitive mechanisms. Consequently, a task design that makes use of categories that *lack* consistent discrete features would strengthen the argument in favor of a sophisticated cognitive classifier.

We designed our test procedure to be much more difficult than those used in past studies of animal categorization. To achieve simultaneous classification, we modified the simultaneous chaining paradigm (or “SimChain,” [41]). In classical SimChain, subjects are presented with a set of stimuli, and are required to touch each stimulus in a prescribed order to receive a reward (see Terrace [42] for review). Our adapted procedure, the *Category Chain*, also requires that subjects touch all four categories in a prescribed order, with different category exemplars used for each trial. This dramatically reduces the rate at which chance responding yields rewards: Touching the stimuli in a random order would earn a reward less than 5% of the time. To complete a trial successfully, subjects must simultaneously represent all four stimuli and classify them into their respective categories, making it much more difficult to solve the task using a simple shortcut. For monkeys to achieve the consistent, high-levels of performance that are described in the literature, they must represent the task cognitively, and not as a simple sequence of associations [41–43].

We also sought to increase the difficulty by trying to improve *stimulus set complexity*. A complex stimulus set must have three characteristics: (1) The set should consist of hundreds of stimuli per category to limit the efficacy of memorization; (2) Stimuli within each category must differ from one another in a variety of ways; and (3) Stimuli across categories must also share similarities. Many studies have pushed one of these boundaries, but few have pressed multiple limits.

Shrier and Brady [13] raised the bar on stimulus set size by testing monkeys using 2,248 images, but only trained a single “human-present” vs. “human-absent” discrimination. Expanding set size augmented both learning speed and peak performance, effects which could not previously be observed with a small number of unique stimuli, and hence a small number of trials. Furthermore, Fagot et al. [44] tested baboons on an “upright” category, and their subjects were only able to successfully transfer to novel stimuli after viewing more than 350 unique stimuli. Apart from these exceptions, few studies have employed large stimulus sets. For example, Wasserman et al. [29] trained only 8 images for each of their 16 categories, and added 4 exemplars per category at a later stage of the study. These numbers are certainly small enough to enable a memorization strategy.

The primary method for ensuring that stimuli are both similar and different has been to use natural categories, such as birds, cats, and flowers. All exemplars will be different, but necessarily similar, and these similarities are believed to be driven by distinct features. The degree of similarity is a crucial factor for experimental manipulation, and feature identification and image manipulation has recently become relatively easy and commonplace thanks to modern computing power.

Researchers have manipulated a wide range of image characteristics, from the easily identifiable and concrete, to the mathematically abstract. In natural photographs of primates, Marsh & MacDonald [4] removed and enlarged eyes, inserted other species’ features, and even removed entire faces. A more abstract study [11] took non-natural cartoon drawings of people and, at differing levels of granularity, scrambled, fragmented, or selectively deleted features of the stimuli, in order to assess the contributions of global and local processing to categorization. At the far end of the spectrum, Smith et al. [45] used only procedurally-generated abstract shapes with quantifiable similarity between stimuli, and manipulated the level of similarity. These studies have produced conflicting results, and it appears that the type of stimuli, be they natural or some form of artificial, may determine the importance of global and local cues for categorization [11]. Moreover, by using artificial stimuli generated entirely from statistical regularities, Smith et al. [45] eliminate any global aspect of the image that might be more than a composition of features, that is, gestalt.

To address limitations in the literature, we used two specific varieties of image categories in this study. In the “photographic” condition, subjects were presented with photographs drawn from four traditional categories of natural images: birds, cats, flowers, and people. These, like the photographic stimuli used in most prior studies, could potentially be classified on the basis of a small number of consistent features, though the stimuli in each category were deliberately numerous and diverse. For example, our “people” category consisted of both color and black-and-white photographs, taken both close-up and at long distances, of both individuals and groups, shot from all angles (including from the back).

In the “painting” condition, subjects were presented with small sections (between 1% and 2%) of paintings by four artists: Salvador Dalí, Jean-Léon Gérôme, Claude Monet, and Vincent Van Gogh. The paintings represent a more abstract problem: stimuli were too small to clearly identify the topic of the painting, and all four painters employed the full color spectrum. Thus, correctly classifying the paintings hinged on more global image properties, such as painterly style.

The relative difficulty of the Category Chain we used stems from two factors: the number of items (four) and the very large number of exemplars on which each category was based. Experiment 1 used thousands of different photographs for each of the four categories. In Experiment 2, each painter’s body of work was represented by hundreds of different samples. Because the exemplars of each item were virtually trial unique in both experiments, subjects would have to compare an indefinitely large number of features of four simultaneously presented stimuli on each trial to represent its ordinal position. Perforce, subjects would have to rely on some sort of *abstract representation of each item*. The ability to form such abstract representations cannot be explained by any current form of association theory.

By using small portions of paintings as stimuli, we sought to challenge feature-based accounts of categorization (e.g. [4, 12]). Individual stimuli within each painting category varied dramatically in terms of their primary image statistics (see the supporting material for more information). As such, category membership could not be defined in terms of discrete features. Successful categorization of the painting stimuli must instead rely on a gestalt appreciation of the image properties. Although such classification could be described in terms of sophisticated statistical learning, it cannot be explained using models of associative strength.

We trained seven monkeys in the Category Chain task. Three learned to classify photographic stimuli, and four learned to classify painting stimuli. We also tested 41 naïve humans, without instruction or any prior experience, on the same Category Chain task, first with photographic stimuli, and subsequently with paintings. The combination of the Category Chain task with our large stimulus sets satisfied both the task complexity and stimulus set complexity requirements needed to rule out the viability of trivially simple or associative classifiers.

## Materials & methods

### Ethics statement

#### Animal subjects

This study was carried out in strict accordance with the recommendations in the Guide for the Care and Use of Laboratory Animals of the National Institutes of Health (NIH). The work was conducted at the Nonhuman Primate Facility of the New York State Psychiatric Institute with permission from its Department of Comparative Medicine’s (DCM) Institutional Animal Care and Use Committee (IACUC), protocol number 200, approved on 09/08/11, and with permission from the Columbia University IACUC, protocol number AC-AAAB1238, approved on 08/10/11.

Subjects were individually housed in rectangular Primate Products Enhanced Environment Housing, each with a nine-square-foot floorspace. Subjects had been previously housed socially, as was standard in the colony, but due to frequent conflict events veterinary staff deemed it unsafe for these monkeys to be socially housed. Regular attempts were made to find new, compatible social partners for these monkeys. Cages were maintained in colony rooms under 12-hour dark and light cycles, and the animals were given access to water ad libitum. Set amounts of Purina Monkey Chow (between 6 and 12 biscuits) and fruit were given after behavioral testing every day. The amounts of food dispensed were determined by the animals’ weight histories; weights were monitored on a weekly basis by research and veterinary staff to ensure subjects stayed at healthy weights. Subjects were given a variety of psychologically enriching tasks to complete at their discretion, beyond those required by behavioral testing. Primate Products enrichment mirrors, puzzle feeders, puzzles tosses, and kong toys were all provided to each individual in their cage; at least once a week, every subject was given sole access to an activity module containing additional kong toys and a prima-swing. No subject was physically harmed or knowingly exposed to potential infection. In accordance with the DCM’s health and safety guidelines, no humans were ever exposed to the monkeys without wearing protective equipment, and as such, the monkeys would not have seen any unmasked human faces. Monkeys were all between 13 and 17 years old during the study. At the conclusion of these studies, the monkeys were assigned to other behavioral studies.

#### Human participants

Human subject protocols were overseen and approved by the Columbia University Institutional Review Board under protocol IRB-AAAA7861, “Cognitive Mechanisms of Serially Organized Behavior”. 41 students enrolled at Columbia University participated in the study to fulfil an introductory psychology class requirement. Students gave written informed consent. No identifying information was collected.

### Experimental task: Category chain

#### Monkeys

As in traditional SimChain [42], Category Chain presents four stimuli on screen at the beginning of each trial, in positions that are randomly assigned. Each trial included one stimulus from each of four categories (e.g., a bird, a cat, a flower, and a human). Monkey subjects had to touch each of these items in a particular order in order to receive a food reward in the form of a banana flavored pellet. Subjects could continue responding as long as they made no errors. However, the first incorrect touch ended the trial, leading to a time-out period and a random rearrangement of the on-screen stimulus positions on the next trial.

What distinguishes the Category Chain from prior SimChain variants is that the specific stimuli for that trial were selected at random from a large image bank. Thus, the cat presented on trial 2 differed from the cat presented on trial 1, and did not appear again for the remainder of the session. In this respect, the specific stimuli are trial unique, and subjects must learn to classify each stimulus according to its respective category before then selecting the stimuli in the correct order.

**Fig 1:**
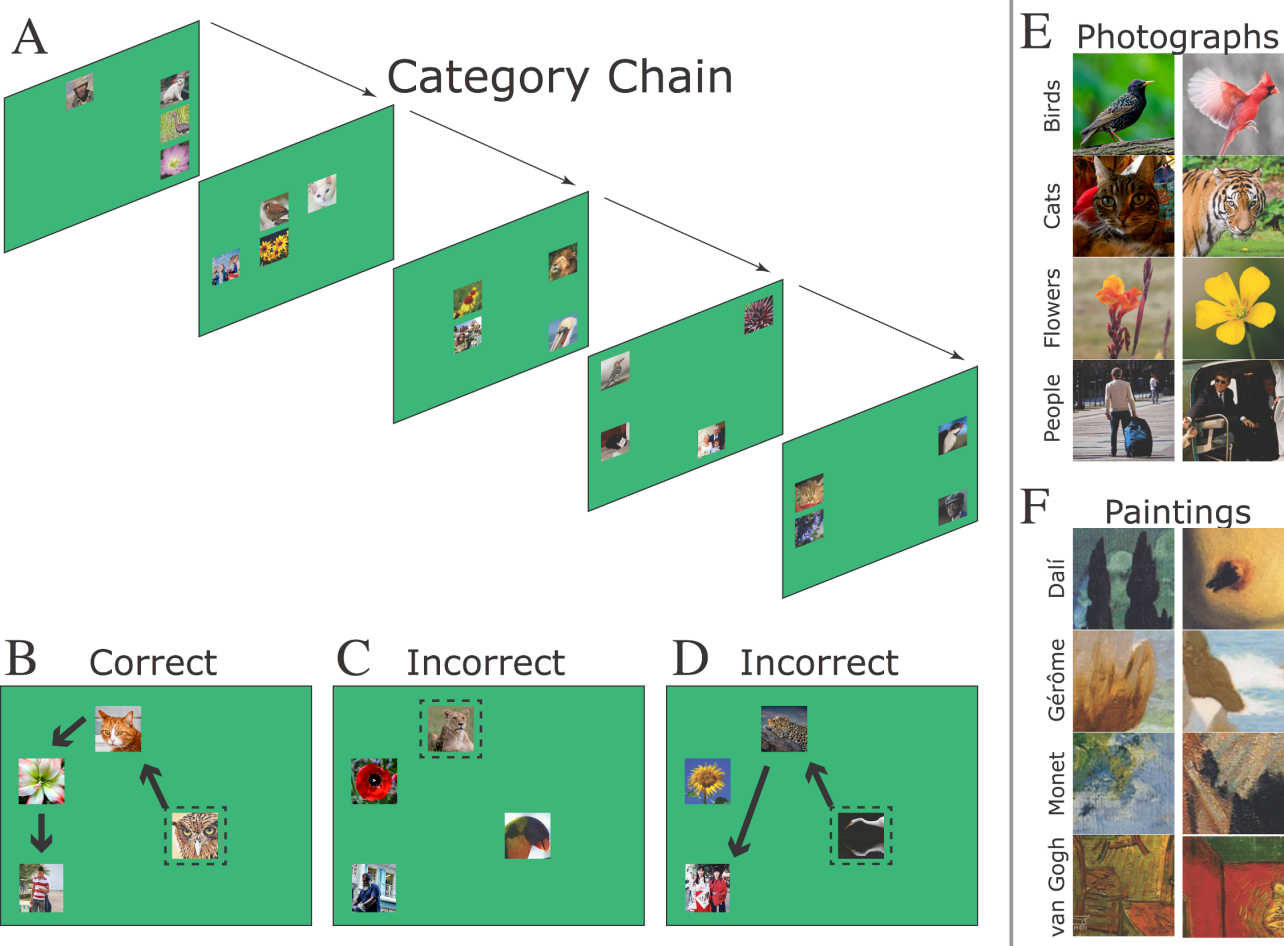
The category chain procedure. **(A)**. Four consecutive trials of the Category Chain task. Each trial presents one stimulus from each of the four categories, but the specific photographs change and the stimulus positions change randomly from one trial to the next. **(B)**. An example of a correct trial. The dashed lines indicate initial touch, and the arrows indicate subsequent touch (neither dashed lines or arrows were visible to subjects). **(C-D)**. Two examples of incorrect trials. In the first case, the initial touch was to the wrong stimulus, so the trial ended immediately. In the second case, the first two responses were correct but the third was incorrect. **(E)**. Two examples each of the four photographic stimulus categories. **(F)**. Two examples each of the four painting stimulus categories.

Fig 1A gives an example of how Category Chain appears over successive trials. Although four stimuli were always presented, each trial used different images. Three example trials are shown in Fig 1. In the correct trial (Fig 1B), each stimulus is touched once, in the order birds→cats→flowers→people. On one incorrect trial (Fig 1C), the initial touch is an error, ending the trial immediately. On another (Fig 1D), a later touch is an error, also ending the trial. Fig 1E and 1F present example stimuli from each of the categories used in the study. Further details about the stimulus sets, including distributions of their primary image statistics, are provided in the supporting information.

Seven rhesus macaques (*Macaca mulatta*) performed the Category Chain task. Three subjects learned to touch stimuli from the photographic categories in a particular order that differed for each subject (Augustus: Flowers → Cats → People → Birds; Coltrane: Birds → Flowers → Cats → People; Lashley: Cats → Birds → People → Flowers). Four subjects learned to put stimuli from the painting categories in order (Benedict: Dalí → Gérôme → Monet → van Gogh; Horatio: van Gogh → Monet → Gérôme → Dalí; Macduff: Monet → Dalí → van Gogh → Gérôme; Prospero: Gérôme → van Gogh → Dalí → Monet). For both photographs and paintings, subjects received extensive training on category membership prior to experiencing the final version of the Category Chain task.

To learn the stimulus categories, subjects were trained using a variant of the Category Chain task. Rather than vary every image during every trial, only a subset of images initially varied. Trials otherwise resembled SimChain: Subjects were rewarded if they touched each image in the correct order. For example, during the first stage of training, the first three stimuli in the chain remained fixed for the duration of the session, but the fourth stimulus varied. For brevity, we will denote each stage of training with a code that indicates which stimuli remained fixed during a session and which varied. “F” denotes a fixed stimulus, and “V” denotes a varying stimulus. Thus, a four-item list in which the first three images are fixed but the fourth varies would be denoted by 1F-2F-3F-4V. All Category Chain sessions lasted 40 trials, and each subject always maintained the prescribed order of categories indicated above.

Training consisted of 15 stages in total. The first four stages varied only one image category at a time (Stage 1: 1F-2F-3F-4V; Stage 2: 1F-2F-3V-4F; Stage 3: 1F-2V-3F-4F; Stage 4: 1V-2F-3F-4F). The next six stages varied two categories at a time (Stage 5: 1F-2F-3V-4V; Stage 6: 1F-2V-3F-4V; Stage 7: 1V-2F-3F-4V; Stage 8: 1F-2V-3V-4F; Stage 9: 1V-2F-3V-4F; Stage 10: 1V-2V-3F-4F). The next four stages varied three categories at a time (Stage 11: 1F-2V-3V-4V; Stage 12: 1V-2F-3V-4V; Stage 13: 1V-2V-3F-4V; Stage 14: 1V-2V-3V-4F). Finally, during the last stage of training, all categories varied, making it a full-blown Category Chain (Stage 15: 1V-2V-3V-4V). Subjects advanced from one stage to the next according to a performance criterion (described below). Once the criterion was met for the final stage, an additional 25 sessions of data were collected, which exhibited subjects’ plateau performance. The results reported in section 4 are based on those 25 sessions.

Subjects learning the photographic categories advanced to the next stage of training when an 80% criterion was met. Subjects learning the painting categories displayed considerably more difficulty with the initial training, which necessitated two changes. First, criterions for advancing to the next stage was lowered to 70% throughout the training. Secondly, the first four stages of training (when only one category varied) were further subdivided into substages that had differing degrees of variation. Rather than varying across the entire image bank, the varying category initially only varied among two items. Once the criterion was met, the number of stimuli used in the varying category was increased to five. This proceeded over successive stages (5, then 10, then 25, then 50, then 100). Once subjects reached the fifth stage of training, these substages were no longer employed.

The housing, operant chambers, and software employed to collect data from monkeys in this study was, unless otherwise specified, identical to that described by Jensen and colleagues [43].

#### Humans

Human participants were given minimal verbal instruction. They were told only (1) that they were to use a mouse to click on images, (2) that feedback would consist green check marks (indicating a correct response) or red crosses (indicating an incorrect responses), and (3) that they should try to get as many correct responses as possible. No mention of either “serial” or “categorical” cognition was made until participants were debriefed.

Participants first completed 120 trials of the Category Chain task using the four photographic categories (using the order Birds → Cats → Flowers → Humans). They then completed 200 trials in which stimuli were derived from works by four painters (Dali → Gérôme → Money → Van Gogh). Unlike the monkeys, participants were given no prior training or instruction regarding the task structure of the category memberships. They had to learn how to categorize the stimuli at the same time as they learned the task demands. Consequently, participants generally began by responding at chance and gradually learned what the correct responses were over successive trials.

## Calculation

In traditional models of choice, each trial consists of a single choice and choice is modeled as the probability of selecting a particular stimulus. The analysis of SimChain (and, by extension, Category Chain), is complicated, however, by the varying number of individual responses during each trial. For example, when presented with a 4 item list, a subject might make one, two, three, or four responses, depending on whether any erroneous responses were made. Consequently, the analysis of Category Chain performance requires the *simultaneous* estimation of a different conditional probability for each response in the sequence.

Let *p*_1_ correspond to the probability of a correct first response, *p*_2_ to the probability of a correct second response, and so forth. The probability of reward in any single trial of the Category Chain depended on four probabilities:

$$p(\text{reward})=p_{1}∙p_{2}∙p_{3}∙p_{4}.$$

For any intermediate degree of progress, the chain of probabilities is cut off following an error (whose probability is (1−*p*_*i*_) for choice *i*). For example, if only the first two responses are correct and the third response is an error, then:

$$p(\text{progress}=2)=p_{1}∙p_{2}∙(1-p_{3}).$$

**Fig 2:**
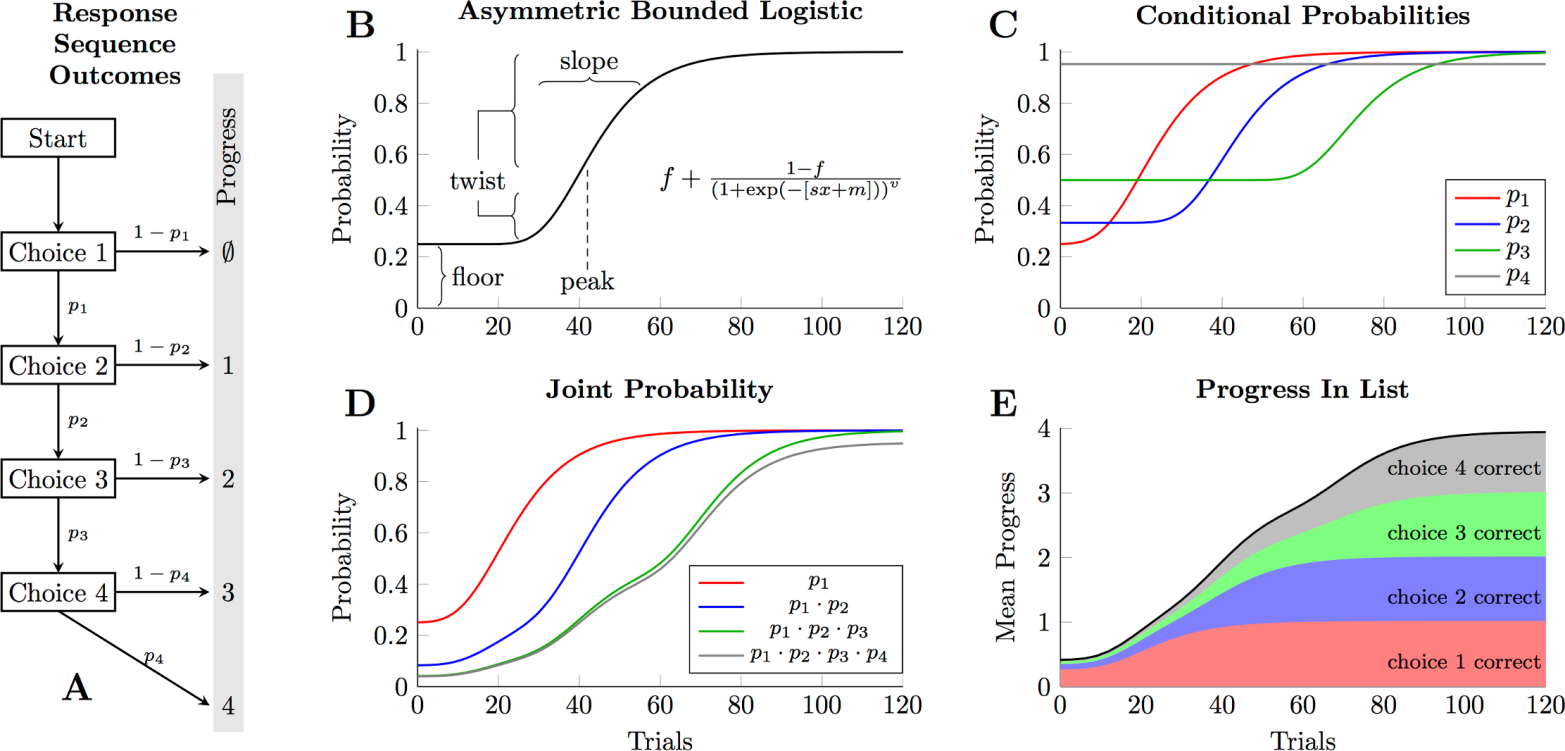
Formal model for describing simultaneous chaining behavior. **(A)**. The decision tree that govern an animal’s “progress” through the list in a single trial. Each choice made by the subject has a probability of being correct *p*_1_, *p*_2_, etc., such that the probability of completing a 4-item list (and thus earning a reward) is (*p*_1_ ∙ *p*_2_ ∙ *p*_3_ ∙ *p*_4_). Since specifying each *p*_*i*_ permits the likelihood to be calculated, posterior distributions for these probabilities can be estimated. **(B)**. The asymmetric bounded logistic function, used to model each choice probability *p*_*i*_ in humans (who lacked prior experience, and so had to learn the categories while doing the task). This function is defined in terms of peak learning rate (governed by *m*), a slope (governed by *s*), a floor term denoting starting performance (governed by *f*), and a twist parameter that influenced the asymmetry in learning speeds early vs. late during learning (governed by *v*). **(C)**. Examples of the conditional probabilities *p*_*i*_ for each of the four choices. Note that *p*_3_ begins higher than *p*_1_ because, should a subject get to the third choice, only two items will remain, making it a 50/50 chance. *p*_4_ is modelled as a constant value near 1.0. **(D)**. Joint probabilities associated with reaching different choice points, using the four probability function in panel C. **(E)**. Average expected progress in the list, computed by taking the sums of the joint probabilities in panel D. Thus, given a function for each choice probability *p*_*i*_, one can also model the progress made by participants.

In order to characterize performance in the Category Chain task, we developed a formal model of subjects’ individual responses. This chain of conditional probabilities is fully depicted in Fig 2A. Performance can be precisely described if each of these probability parameters can be estimated. Because the monkeys received extensive training prior to test, their performance had already achieved a stable ceiling. Consequently, the probability of a correct choice to stimulus *i* was estimated using the logit link:

$$p_{i}=\frac{1}{1+exp(-m_{i})}$$ (Eq 1)

Here, *m*_*i*_ corresponds to the intercept term of a logistic regression, and as such governs the probability of a correct response *p*_*i*_ for subject *i*.

The human data presented a more complicated analysis problem, however, because they learned to classify the stimuli over the duration of their participation. Thus, each probability was expected to begin at chance, then grow toward 1.0 over time. To characterize learning, we used a variant of the generalized logistic function [46], which we call the asymmetric bounded logistic:

$$p_{i}(t)=f+\frac{1-f_{i}}{{\left(1+exp(-[s_{i}∙t+m_{i}])\right)}^{v_{i}}}$$ (Eq 2)

Here, *p*_*i*_(*t*) refers to the probability of a correct response to stimulus *i* at time *t*. The function then accepts four parameters. The slope (*s*) influences the speed at which learning unfolds, and the peak (*m*) influences when learning begins to differ from chance. The level of chance performance for each stimulus is in turn governed by a floor parameter (*f*). Finally, a twist parameter (*v*) is included because past empirical work on SimChain suggests that once learning begins, it improves very rapidly at the outset, with diminishing returns as performance reaches asymptote [43]. A depiction of the asymmetric bounded logistic is presented in Fig 2B, along with a description of the contributions of each parameter.

Fig 2C shows hypothetical learning curves for each response over the course of 120 trials. The logistic functions for *p*_1_, *p*_2_, and *p*_3_ all have the same parameters for slope (*s* = 0.1) and twist (*v* = *e*^6^), but differ in their values for the floor *f* and the peak *m*. The floors differ because, when a participant uses process-of-elimination search, chance performance given *n* stimuli is $\frac{1}{n}$ for the first item (because there is a 1 in *n* chance of chosing the correct alternative), $\frac{1}{n-1}$ for the second item, and so forth. The intercepts differ because progress cannot be made on discovering the identity of Item 2 until some learning regarding Item 1 has begun. Finally, the last probability *p*_4_ is a constant close to 1.0 because subjects almost never make mistakes on the final choice, again thanks to process-of-elimination search.

The need for asymmetry in the learning rate (with a long period at some floor value, followed by a sharp acceleration and then gradual diminishing returns) stems from SimChain’s process of sequential discovery. Prior to discovering the identity of the first stimulus, subjects cannot gain information about the remaining stimuli. It is only after the first stimulus was selected with some consistency that any information about later list items can be discovered. This is why the conditional probability for *p*_3_ remains flat for 50 trials in Fig 2C. It is only after the first two stimuli have been acquired that the subject has an opportunity to perform above chance with respect to the third stimulus.

Fig 2D depicts the joint probability of the same participant making progress through the list. The red line is the same as in Fig 2C, depicting how often the first choice is correctly made. The blue line, however depicts how often the participant got at least two choices correct (determined by multiplying *p*_1_ and *p*_2_), the green line at least three correct, and so forth. Fig 2E depicts the mean expected progress in the list, and is determined by taking the sum of the joint probabilities in Fig 2D. In this case, the participant begins at chance levels (progress = 0.4167), but is earning a reward for almost every trial by trial 120.

Parameter estimates were obtained using the Stan language [47]. The analysis script is included as supporting information.

## Results

Our results provided compelling evidence of category learning of photographic and painterly stimuli by monkeys that could not be explained by association theory. Similar results were obtained from human subjects trained to respond to the same stimuli.

We analyzed the behavior of monkeys and humans performing the Category Chain task with respect to four photographic categories and four painting categories. In particular, our analysis focused on making estimates of the conditional probabilities of the responses to each stimulus, and their corresponding reaction times.

### Monkeys

**Fig 3:**
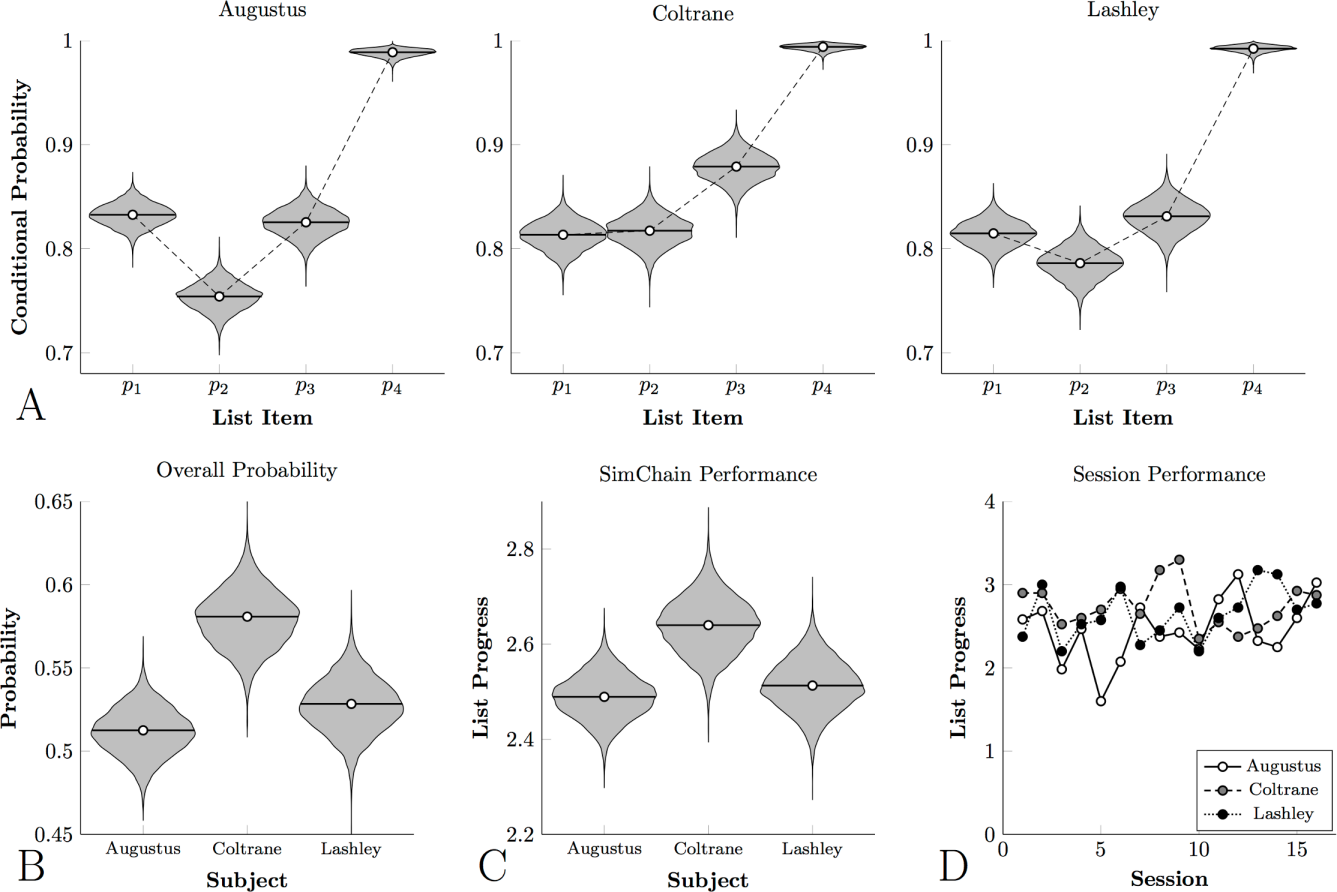
Monkey Category Chain performance for photographic stimuli. In all cases, violin plots represent the posterior density for the parameter estimate. **(A)**. Conditional response probabilities for each subject with respect to each list item. **(B)**. Overall probability that any given trial will result in reward delivery (equal to the product of the conditional probabilities) for each subject. **(C)**. Mean progress in the list on each trial for each subject, averaged across sessions. **(D)**. Observed mean progress in the list on each trial for each subject, for each of the last 16 sessions.

Fig 3A depicts the conditional probabilities for the three monkeys who learned to classify photographic stimuli. Fig 3B depicts their overall probability of obtaining a reward and Fig 3C depicts their mean progress in the list. The violin plots correspond to the posterior distribution of each estimate. Despite the presentation of different stimuli during every trial, accuracy was reliably high. Subjects responded correctly to at least 75% of the first-position stimuli (as compared to chance accuracy of 25%); at least 83% of the second-position stimuli (chance accuracy = 33%); and at least 85% of third-position stimuli (chance accuracy = 50%). Jointly across trials, this led to rewards being earned on roughly half the trials. Fig 3D shows mean SimChain performance for each participants’ last 16 sessions, demonstrating the stability of their performance.

**Fig 4:**
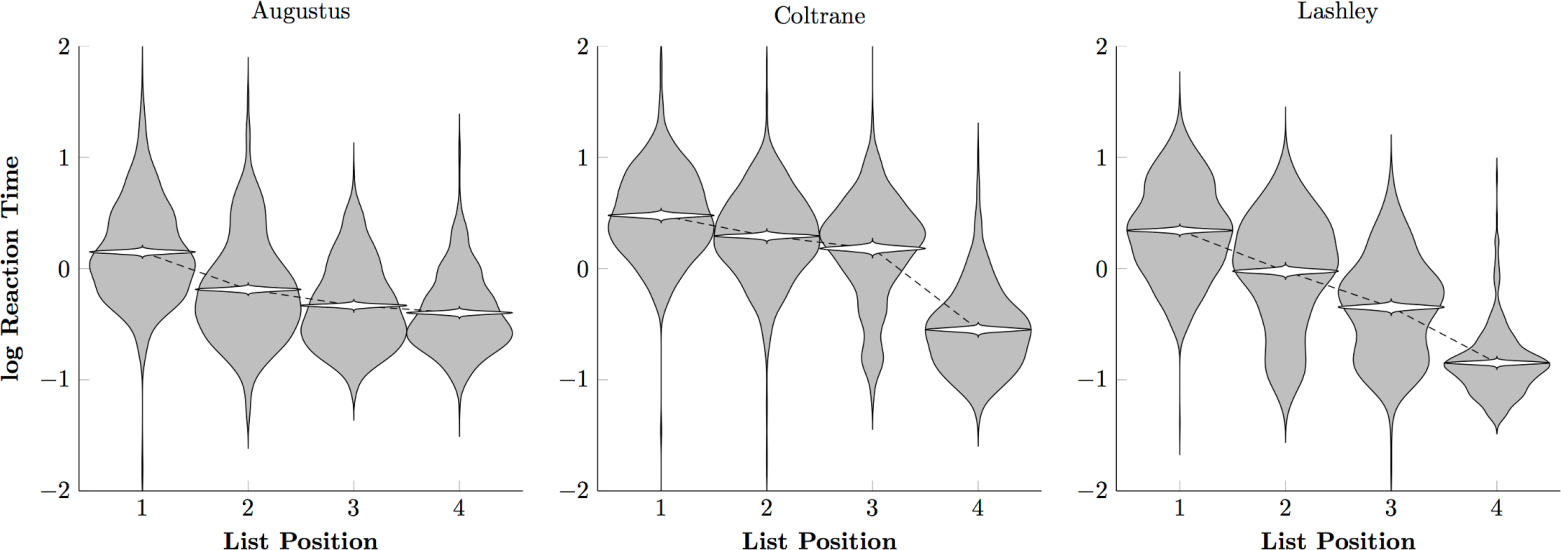
Monkey reaction times to photographic stimuli on a log scale. Violin plots show the distribution of log reaction times (in gray) and the credible interval for the mean reaction time (in white) for each monkey.

Fig 4 depicts the log reaction times of the photographic monkeys, displaying both the overall variability of reaction times (gray violins) and the credible interval for the mean (white violins). Subjects were consistently accelerated their responding over the course of a trial, as would be expected using of a process-of-elimination strategy.

**Fig 5:**
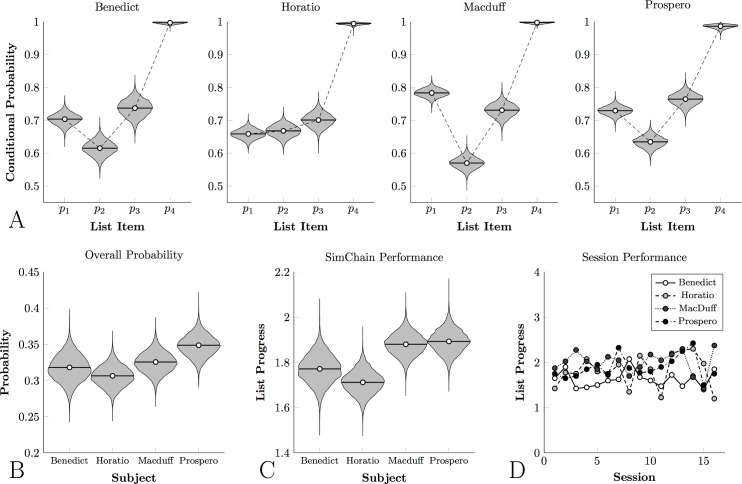
Monkey Category Chain performance for painting stimuli. In all cases, violin plots represent the posterior density for the parameter estimate. **(A)**. Conditional response probabilities for each subject with respect to each list item. **(B)**. Overall probability that any given trial will result in reward delivery (equal to the product of the conditional probabilities) for each subject. **(C)**. Mean progress in the list on each trial for each subject, averaged across sessions. **(D)**. Observed mean progress in the list on each trial for each subject, for each of the last 16 sessions.

Fig 5A depicts the conditional probabilities, Fig 5B depicts overall probability, and Fig 5C depicts mean performance for the four monkeys who classified painterly stimuli. Although all four animals displayed conditional probabilities above chance, the overall accuracy was consistently lower than that observed for photographic classification. As a result, only about one trial in three ended with reward. This corresponded with consistently lower performance on a session-by-session basis than subjects who classified natural photographs, as can be seen in Fig 5D.

**Fig 6:**
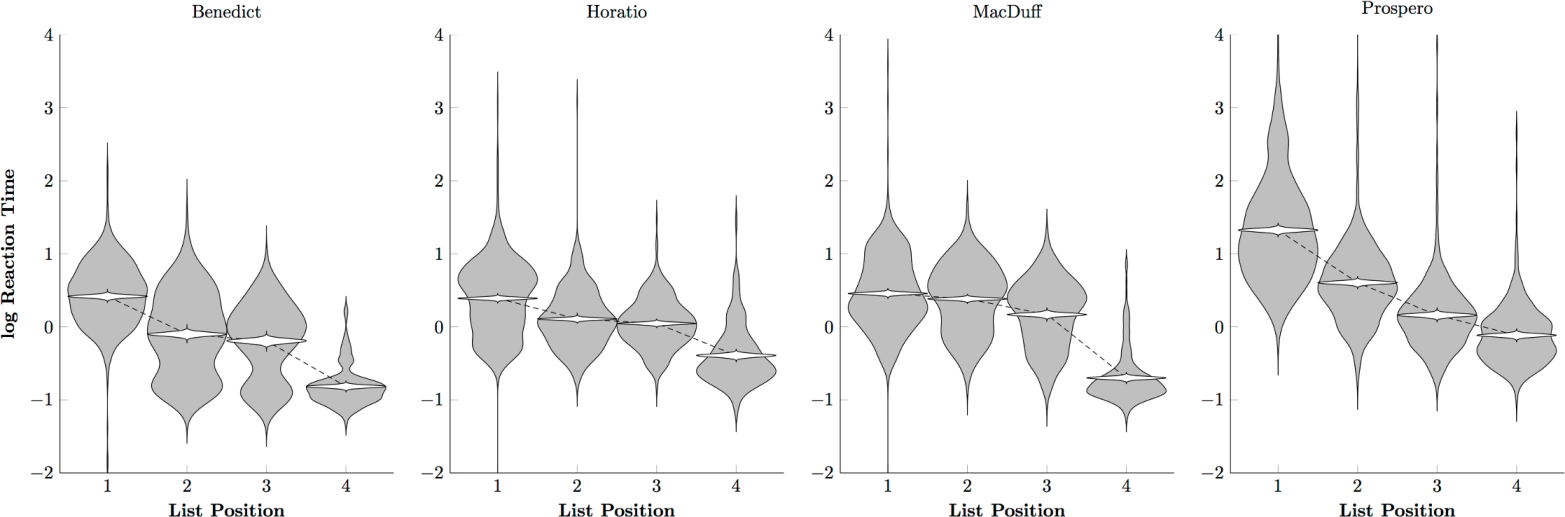
Monkey reaction times to painting stimuli on a log scale. Violin plots show the distribution of log reaction times (in gray) and the credible interval for the mean reaction time (in white) for each monkey.

Fig 6 tells a similar story about the log reaction times: Subjects responded more rapidly to later list items than to earlier ones, but tended to respond more slowly overall. One animal in particular, Prospero, tended to respond at only about ^1^/_3_ as rapidly as did the photographic group (cf. Fig 4). This suggests that the painterly stimuli were more difficult to classify, relative to the comparatively automatic classification of the photographic stimuli.

Overall, these results resemble patterns of learning observed in classical SimChains presenting fixed arbitrary photographs [41, 43], and in SimChains for which the list order was determined by some psychophysical dimension [48]. This demonstrates both that subjects were able to classify the categorical stimuli, and that the serial learning of categories was consistent with other previously reported forms of serial learning. What distinguishes the current task from previous studies of serial learning (e.g. using classical SimChain) is that every stimulus changed on every trial. This meant that subjects had to classify the stimuli before they could determine their serial order. Additional details, including distributions of estimated parameters, are provided in the supporting information.

### Humans

**Fig 7:**
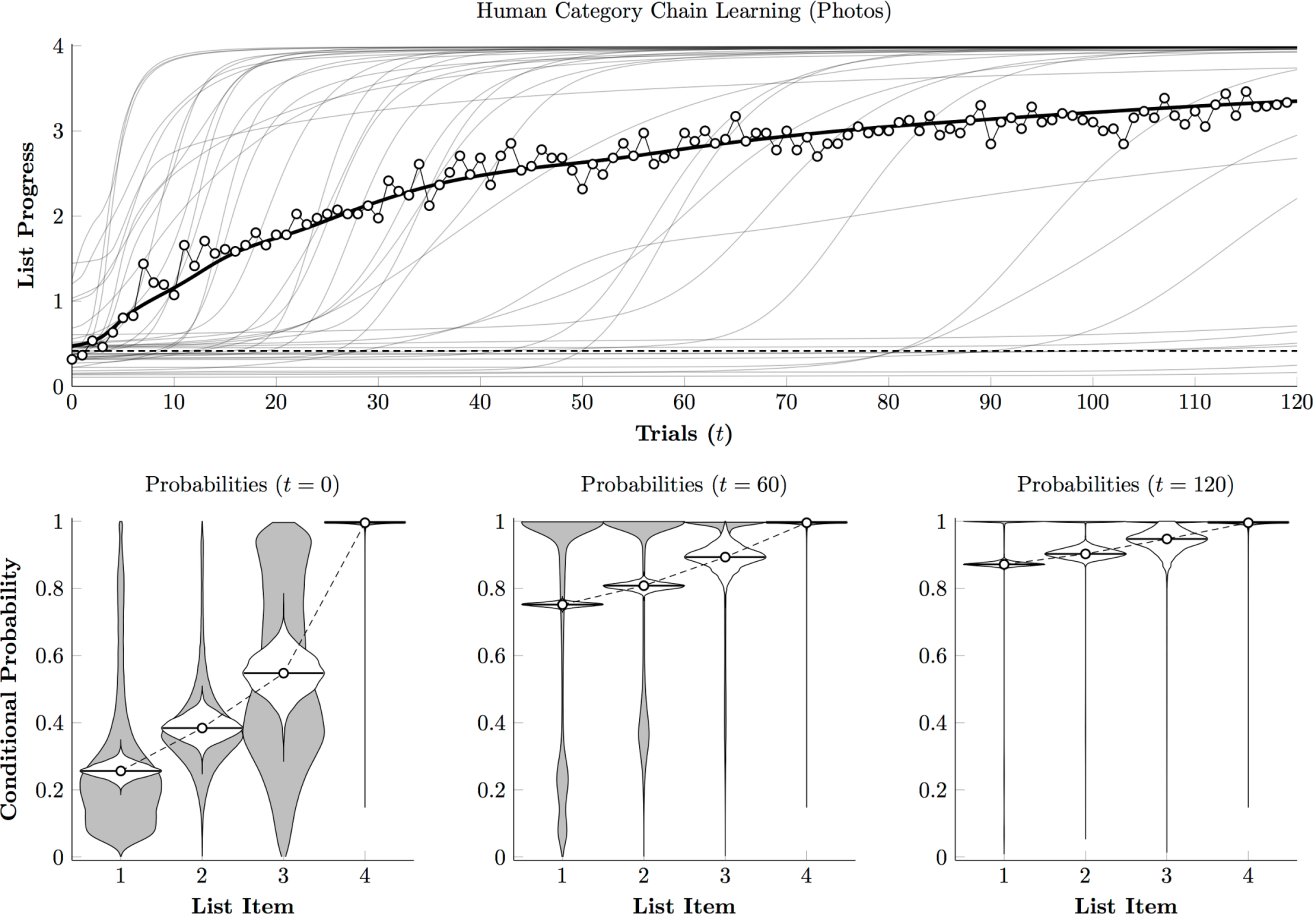
Human performance estimate for photographic stimuli. **(Top)**. Estimated time course of Category Chain performance for individual participants (gray curves) and the overall mean (black curve). White points correspond to the empirical mean of performance across subjects on each trial. Chance is indicated by the dashed line. **(Bottom)**. Estimated conditional response probability for each list item at session onset (*t* = 0), midway through the session (*t* = 60), and after the last trial (*t* = 120). Violin plots show the distribution of individual probability estimates (in gray) and the credible interval for the mean probability across participants (in white). Note that mean probabilities are usually much lower than the population mode (which is near 1.0), due to a subset of participants who remain at chance throughout training.

Fig 7 (top) depicts the best-fitting learning curves, using Eq 2, of individual human participants during the photographic phase (semitransparent lines), as well as their group mean (solid black line). Additionally, the empirical trial means are plotted as white points. These learning curves indicate that most participants reached near-perfect performance (classifying all four stimuli correctly on nearly every trial) within the first 40 trials of 120. The steep learning curves displayed by most participants suggests that once the task demands were understood, discovering the order unfolded very rapidly.

Fig 7 (bottom) confirms this suspicion, plotting the population density function for the conditional probabilities (gray violins) as well as the posterior distributions for mean performance. Performance at the start of training (*t* = 0) looks just as one would expect for chance performance, but by the session’s midpoint (*t* = 60), a bimodal distribution had emerged of predominantly successful participants with a handful still responding at chance levels. By the end of the session, the bimodal data continue to drag the overall mean down somewhat, but the mode is so close to 1.0, as the participants who are making close to zero errors dominate the distribution. The rapid acquisition by humans is consistent with an account whereby they identify the categories using familiar linguistic labels.

**Fig 8:**
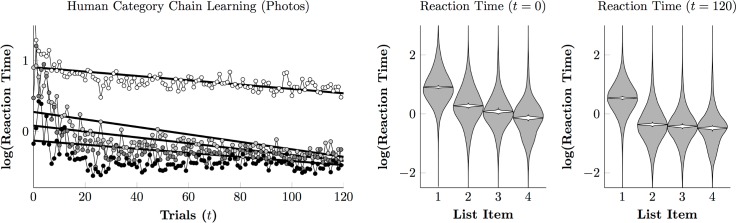
Human reaction times to photographic stimuli on a log scale. **(Left)**. Regression model of estimate first touch (white), second touch (light gray), third touch (dark gray), and final touch (black). **(Right)**. Estimated reaction time at session onset (*t* = 0) and after the last trial (*t* = 120) for touches to each list item. Violin plots show the distribution of log reaction times (in gray) and the credible interval for the mean reaction time (in white).

Fig 8 tells a similar story with regards to the reaction times. Fig 8 (left) depicts the empirical means of the log reaction times as points for first responses (white points), as well as the second (light gray), third (dark gray), and fourth (black) responses. With the exception of very early trials, these were approximately linear, so a hierarchical linear regression was performed (black lines). These results suggest that, at the outset of training, responses displayed the characteristic acceleration seen in monkeys, in which each response was made more quickly than the last (compare to Figs 4 and 6). This pattern is consistent with a visual search strategy, working by process of elimination one response at a time. However, by the end of the session, a different pattern had emerged: A long interval for the first responses, followed by rapid selection of the second through fourth items. This pattern is more characteristic of a plan-then-execute approach. This pattern is also evident in Fig 8 (right), which shows the posterior population estimates (gray violins) and credible intervals for the line of best fit (white violins) at both the start (*t* = 0) and the end (*t* = 120) of training. Estimates showed a consistent downward trend at the start of training. Reaction times at the end of training were very similar after the first response.

**Fig 9:**
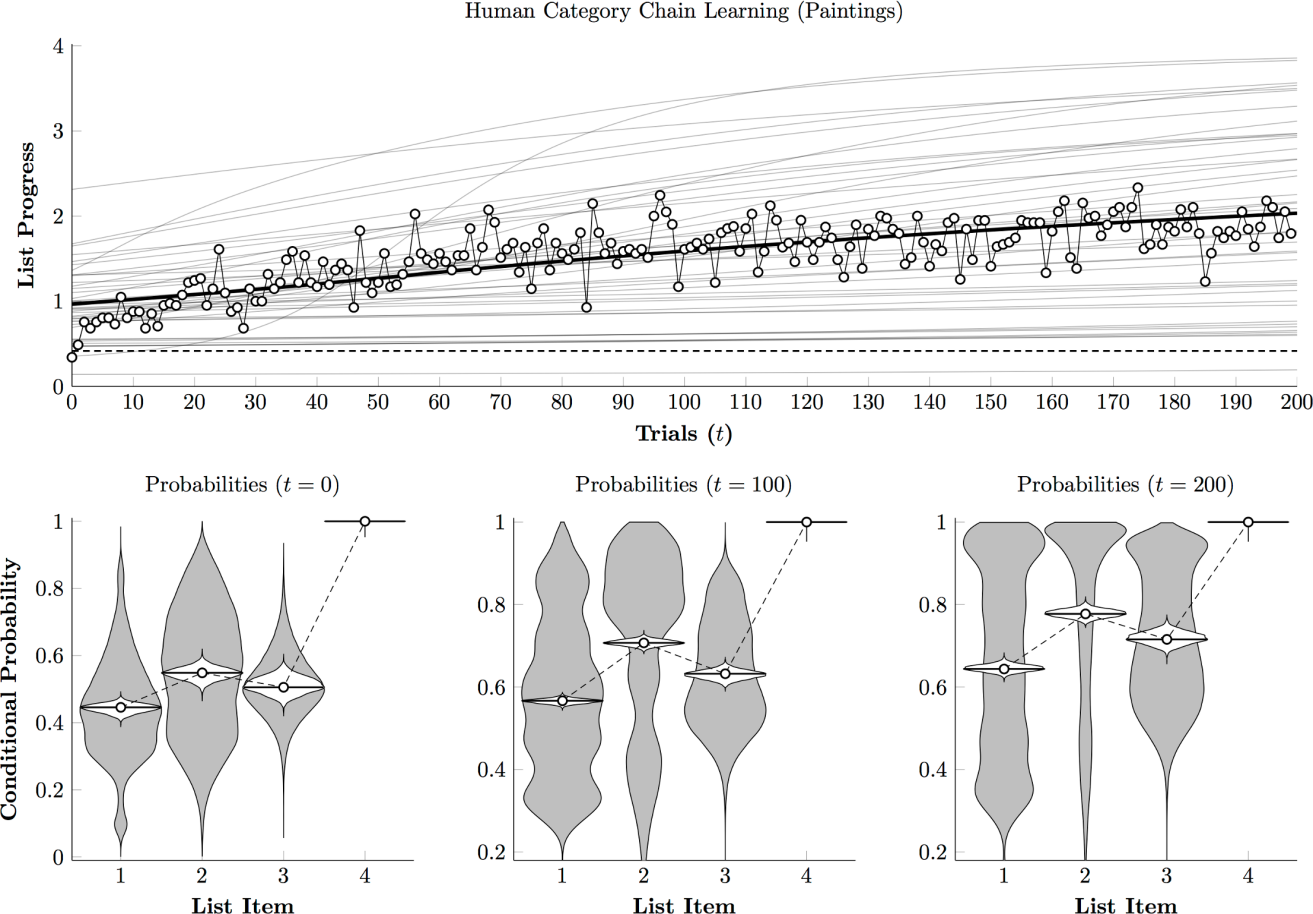
Human performance estimate for painting stimuli. **(Top)**. Estimated time course of Category Chain performance for individual participants (gray curves) and the overall mean (black curve). White points correspond to the empirical mean of performance across subjects on each trial. Chance is indicated by the dashed line. **(Bottom)**. Estimated conditional response probability for each list item at session onset (*t* = 0), midway through the session (*t* = 100), and after the last trial (*t* = 200). Violin plots show the distribution of individual probability estimates (in gray) and the credible interval for the mean probability across participants (in white).

Fig 9 (top) depicts the performance of the same participants, in terms of Eq 2, to the painting phase of the experiment. Unlike Fig 7, participants did not show rapid acquisition, instead improving only gradually as the session unfolded. Similarly, Fig 9 (bottom) shows that the population distributions of conditional probabilities do not converge rapidly toward ceiling as they did in the photographic condition. Although participants did tend to improve to varying degrees (including a few who were able to classify all four stimuli), many improved so slowly as to be indistinguishable from chance.

**Fig 10:**
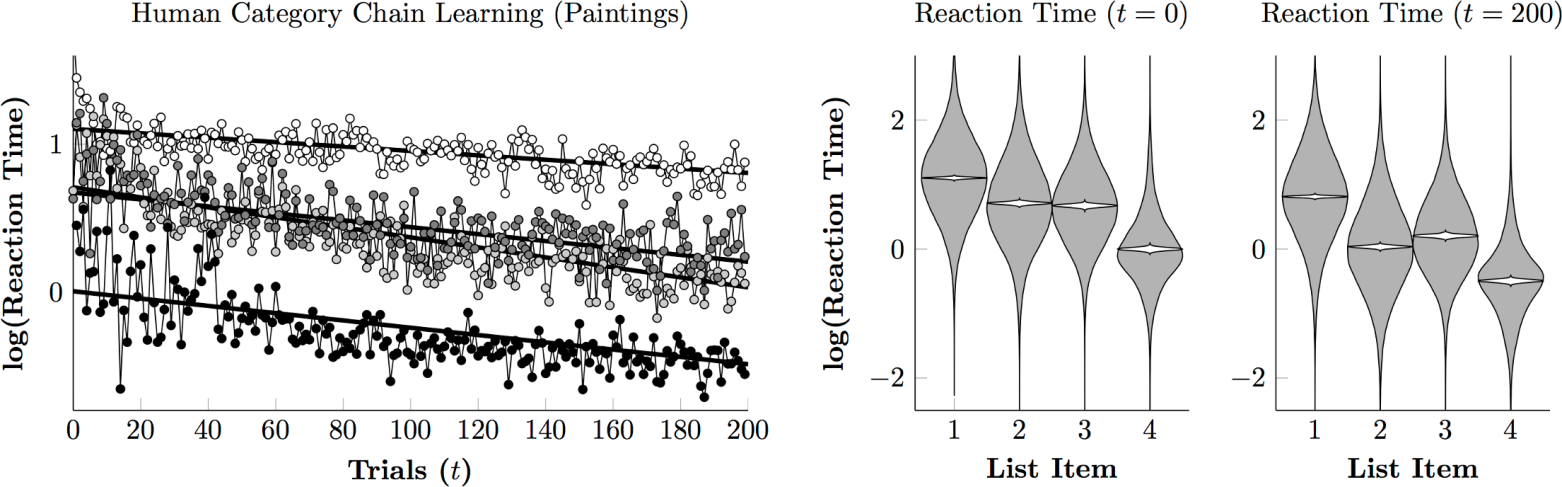
Human reaction times to painting stimuli on a log scale. **(Left)**. Regression model of estimate first touch (white), second touch (light gray), third touch (dark gray), and final touch (black). **(Right)**. Estimated reaction time at session onset (*t* = 0) and after the last trial (*t* = 200) for touches to each list item. Violin plots show the distribution of log reaction times (in gray) and the credible interval for the mean reaction time (in white).

Fig 10 (left) depicts reaction times over the course of the painting phase. A plan-then-execute approach is no longer evident, with reaction times for the second and third items remaining consistently long and similar to one another. The third response (in which participants must distinguish Monet from van Gogh) appeared especially difficult, emerging as slower than the second response by the end of the session.

Overall, these results provide a dramatic contrast. In the photographic phase, despite receiving no verbal instruction, participants were able to determine (1) that stimuli belonged to regular categories, (2) that rewards depended on selecting categories in a particular order, and (3) to classify stimuli consistently. Within 60 trials, most previously-naïve participants were classifying stimuli with near-perfect accuracy (Fig 7), whereas highly trained monkeys performing the same task and evaluating the same stimuli only managed to earn rewards on about 50% of trials (Fig 3). In the painting phase, however, humans demonstrated much greater difficulty identifying the categories and thus classifying the stimuli, despite being familiar with the task. By the end of 200 trials, humans (Fig 9) still resembled monkeys (Fig 5), classifying the first three items with approximately 70% accuracy.

## Discussion

Our results compare performance on two primate species, macaques and humans, across two types of visual categories: naturalistic photographs and famous artists’ paintings. Category exemplars were selected from large and highly disparate sets of images. Accuracy during the painting phase was lower for both monkeys and humans, indicating that the paintings were more difficult to categorize. Nevertheless, both species were able to successfully learn and simultaneously classify stimuli from both sets of categories.

This categorization is noteworthy for two reasons. First, our task (the Category Chain) was unusually demanding, requiring that subjects simultaneously classify four stimuli by responding to four different response locations on each trial. Second, both the number of photographic and painting stimuli were large and diverse. The paintings, in particular, could not be categorized by attending of a handful of discrete features. These two factors (task complexity and stimulus set complexity) jointly provide compelling evidence that subjects processed stimuli as gestalt percepts rather than resorting to a simple feature-based strategy. These percepts permitted subjects to classify stimuli even when category membership depended on high-level stimulus properties rather than low-level image statistics or specific feature discriminations. Stimuli in the painting categories lack clearly definable features, and this is a problem for associative models (which require features with which rewards can be associated to explain behavior).

The photographs are representative of ecological stimuli; humans are familiar with these categories, and we possess unambiguous linguistic scaffolds for each. Although captive monkeys have little exposure to wildlife and do not possess language, primates nevertheless evolved in the presence of birds, flowers, cats, and other primates, and past experiments have demonstrated a greater aptitude at classifying naturalistic photographs than those of man-made objects [49].

While our monkeys performed well above chance with photographic stimuli, they made consistent, systematic errors, even after thousands of trials of training. By comparison, most human participants were able to perform the task with close to zero errors after fewer than 100 trials. Many studies have reported that monkeys tend to make errors, even when the discriminations are simple and the animals experienced. One hypothesis for this persistent error rate is that while both monkeys and humans face a speed-accuracy tradeoff, monkeys tend to favor speed while humans tend to favor accuracy [15, 50]. This does not seem like a satisfactory account of the present data, however, because monkeys and humans had similar reaction times by the end of training (Fig 4 vs. Fig 8, Fig 6 vs. Fig 10). Another possible reason for differences in response accuracy may stem from species differences in executive control. Monkeys tend in general to behave under the influence of stimuli, whereas humans tend to behave under the influence of more abstract task demands [51–52]. Because the photographic stimulus categories were familiar to human participants, recognition appeared fairly automatic and was unperturbed by changes in the stimuli from trial to trial. If the monkeys, however, deliberately assessed categorical membership of the stimuli on every trial, it would help explain persistent errors.

The paintings are not representative of any stimuli that are frequently encountered in the wild and were more difficult to classify. When classifying the paintings, monkeys were less accurate and their systematic errors were more frequent. Humans also found the paintings phase more difficult, making more errors and learning more slowly (Fig 7 vs. Fig 9). Nevertheless, the monkeys again received thousands of trials of training, yet by the end of 200 trials, human performance plateaued, and participants were about as accurate as the monkeys.

Apart from Augustus, the monkeys did not display markedly different reaction times to paintings than they did to photographs. This suggests that most monkeys used a similar classification strategy with the paintings stimuli as with the photographic stimuli. Unlike the monkeys, humans displayed two patterns of response. The photographic stimuli elicited a “single slow response, followed by three quick responses” pattern (consistent with planning an entire sequence before executing the plan), whereas the painting stimuli forced participants to pause at each decision. In this respect, humans differed from monkeys when classifying photographs, but resembled them when classifying paintings. The differing patterns of reaction times and the very slow improvement in performance suggest that human participants had no existing linguistic representations to link to these percepts. Participants may have seen full images of some of the paintings before, but by zooming in on the details and brushwork of the paintings, we attenuated whatever benefits would have come with familiarity with the artists’ works. When faced with sufficiently abstracted categories, humans seem to rely on a different, pre-linguistic classifier, which we evidently share with other primates.

One of the chief difficulties in determining how features are used by classifiers is that it is unclear what constitutes a “feature” in objective terms. For example, the plausibility of the hypothesis that photographs are classified based on discrete features (such as eyes and beaks) depends on the ease with which we are able to identify such features. Traditionally, features were either defined in terms of “bottom-up” or “top-down” information [53]. A bottom-up classifier identifies a feature through the assemblage of low-level sensory features (such as on-center receptive fields and edges). Such systems are thus strictly driven by primary sensory information, without any influence of prior expectation. Top-down classifiers, on the other hand, are heavily influenced by prior knowledge, and act on sensory representations that have already undergone several rounds of synthesis and integration. It is very difficult to provide direct evidence of top-down processing in animals [54], and as a result the study of animal classifiers has focused almost exclusively on bottom-up processing [4, 12, 55]. Although bottom-up processing is clearly involved in how the brain classifies images, it is far from clear that it is *sufficient* to explain the abilities of non-human animals.

### Machine learning comparison

In order to compare our empirical data to a purely bottom-up approach, we simulated task performance using two classifier algorithms. First, the “bag-of-features” (BoF) image classifier. This sophisticated algorithm makes discriminations only on the patterns of low-level statistical regularities. The algorithm collects salient features without incorporating order, structure, or spatial information, yet despite this simplicity, the BoF classifier achieves high performance in a variety of imaging applications [56]. Insofar as subjects outperformed the algorithms, we take this as evidence that subjects’ strategy was more than merely bottom-up, instead involving at least some hierarchical processing.

Second, we used AlexNet, a well-known convolutional deep learning network (DLN) that has prior training with photographic classification. In contrast to bag-of-features, a convolutional DLN is explicitly inspired by cortical processes. Its lowest level of image processing compares the image to a series of linear filters (such as “light on the left, dark on the right”), similar to orientation and edge detection in primary visual processing. These low-level filters make weighted contributions to the firing of nodes at a higher level, with convolutional connectivity between layers. As a result, the second layer can resolve somewhat more complex forms than the first layer. With each additional convolutional layer, more and more complex stimulus features can be identified. We made use of the five convolutional layers in AlexNet, as well as two subsequent fully-connected layers, which is sufficient for arbitrary photograph identification [57]. Because AlexNet comes pre-trained to classify images in general, its off-the-shelf image classification capabilities are very strong, and appear similar to the capabilities of higher order areas of the monkey visual system [58].

In both the case of the BoF algorithm and the DLN algorithm, training on our stimulus categories followed the traditional practice of splitting each stimulus set into a training set and a validation set, using the training set to educate the algorithms, and then producing a classification matrix of conditional probabilities (e.g. “odds of identifying stimuli from each image category as a cat”). This was particularly fast using AlexNet, because its network comes pre-trained on a vast array of photographic images. Consequently, rather than having to retrain AlexNet from scratch, we needed only to identify which nodes in a particular layer of processing were predictive of our stimulus categories using a support vector machine (SVM).

To accomplish this, multiple iterations of training and validation were performed. For each iteration, 30% of the stimuli in each category were designated as a training set, and the remaining 70% were later used as a validation set. Additionally, rather than basing categorization on AlexNet’s output layer, categorization was inferred by extracting a predictive signal from lower layers of the network. Using MATLAB’s Statistics & Machine Learning Toolbox (Mathworks, version 2017a), a SVM identified patterns of activation among networks nodes that were predictive of the categories specified for the training set. Then, this SVM categorized each stimulus in the validation set. This approach has the advantage that it allows us to specify categories of our own, rather than relying on the categories AlexNet was originally trained with. Since categorization accuracy varies as a function of which stimuli are used for training and validation, we repeated the process (with random assignments of stimuli as training vs. validation) 500 times. We report that average performance across those many iterations. The supporting materials include a MATLAB script that performs both of these analyses.

**Fig 11:**
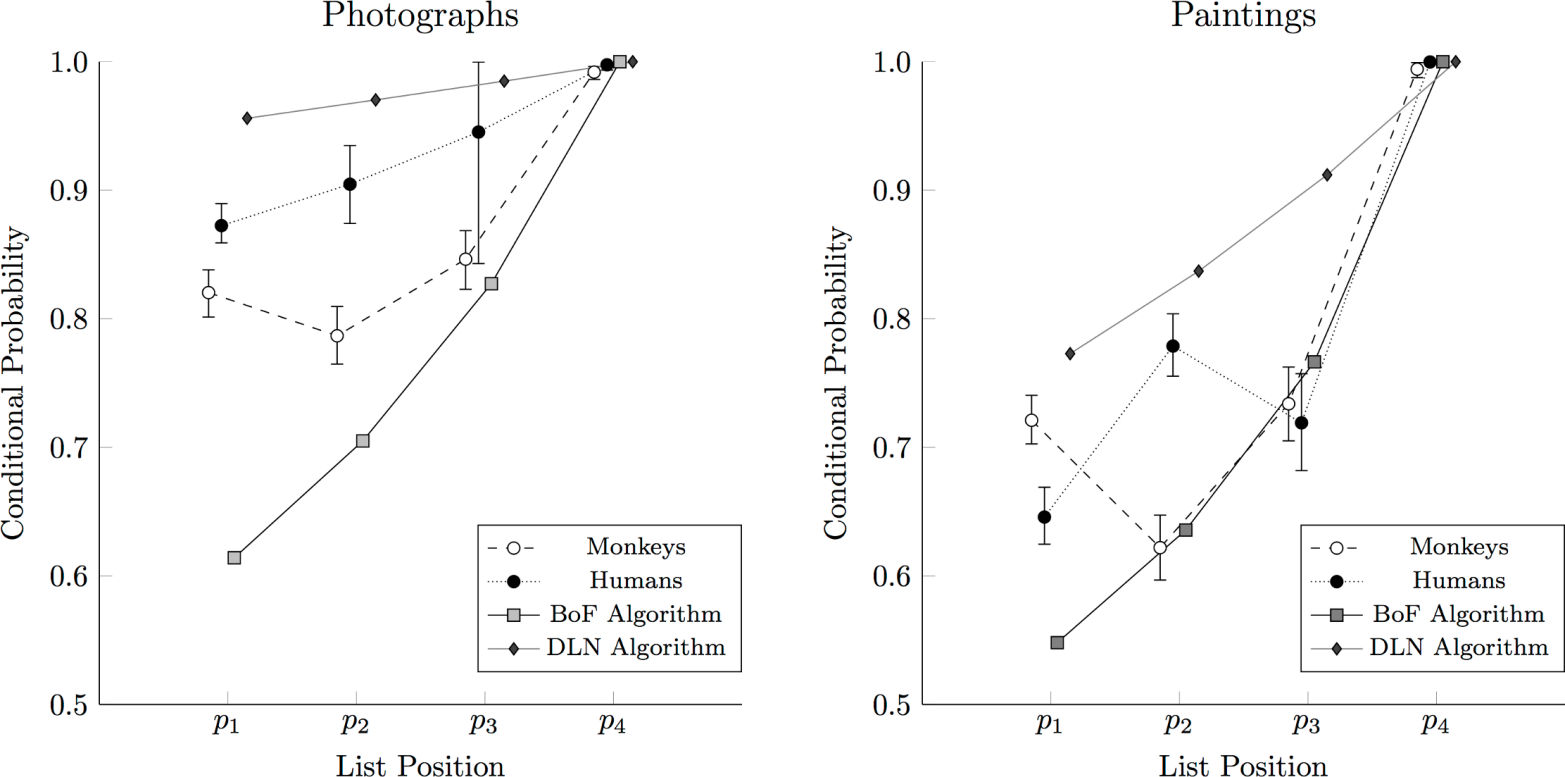
Correct response probabilities for monkeys, humans, and a computer vision algorithm. Estimated response probabilities for monkeys (white points) and humans (black points) for each of conditional probabilities *p*_1_ to *p*_4_ include error bars for the 95% credible interval of the estimate. Accuracy is also included for the bag-of-features (BoF) classification algorithm (gray squares) and AlexNet deep learning network (DLN) (dark gray diamonds). **(Left)**. Performance in classifying stimuli from the four photographic categories. Humans systematically outperformed monkeys, who in turn outperformed the BoF algorithm, but neither group outperformed the DLN algorithm. **(Right)**. Performance in classifying stimuli from the four painting categories. Neither humans nor monkeys were consistently superior, although while both outperformed the algorithm on the difficult initial discrimination, again, neither outperformed the DLN algorithm.

Fig 11 shows the mean probability of a correct response for monkeys (in white circles), humans at the end of one session (in black circles), the BoF algorithm (in gray squares), and the DLN algorithm (in dark grey diamonds) when presented with both photographs and paintings. The algorithm probabilities assume a process-of-elimination search with no backwards errors. Of particular importance was the probability *p*_1_ of selecting the first item correctly (where chance performance would be 0.25), since this was the case in which the target had to be distinguished from three distractors. For the photographic stimuli, humans clearly outperformed monkeys, but monkeys also outperformed the BoF algorithm. The paintings, however, yielded lower performance for all cases, confirming the intuition that the paintings ought to be more difficult to differentiate. In addition, although humans and monkeys outperformed the bag-of-features algorithm on the first painting discrimination, neither species clearly outperformed the other. On both photographs and paintings, the DLN algorithm outperformed monkeys, humans, and the BoF algorithm.

**Fig 12:**
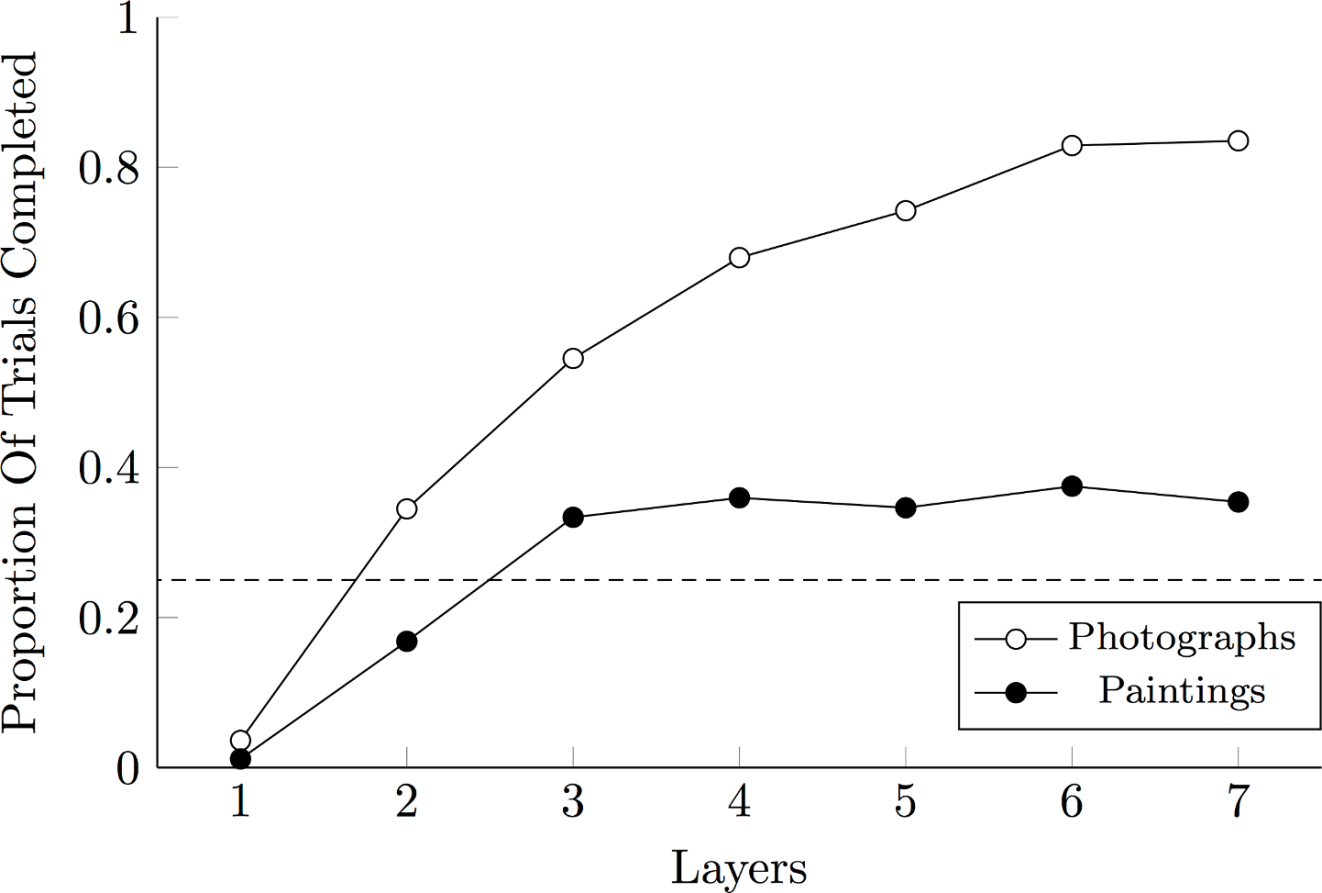
Category Chain performance by AlexNet, divided by layer. Proportion of Category Chain trials completed (with all four stimuli categorized successfully), based on the categorizations of separate layers of AlexNet, whether these layers be convolutional (1 through 5) or fully connected (6 & 7). The dashed line indicates chance performance.

To investigate differences how AlexNet processed these stimuli, we performed image classifications using SVMs at each of AlexNet’s five convolutional layers, and both of its fully connected layers. The result of this analysis, presented in Fig 12, suggests that although high-level structures emerged with each additional layer when categorizing the photographs, the same cannot be said of the paintings.

Classification was initially attempted using only the first convolutional layer, which could only complete the Category Chain on 3% of trials for the photographs and only 1% of the trials for the paintings. When classification was based on the second convolutional layer, however, overall performance rose to 34% for photographic chains and 16% for painterly chains; performance using the third layer rose further to 54% for photographs and 33% for paintings. With each additional layer, photographs continued to improve, until 84% of Category Chains were successfully completed using the second fully connected layer (thus also depending on the five preceding convolutional layers). However, the categorization of painterly stimuli showed no improvement past the third layer: Despite five convolutional layers and two fully connected layers acting as classifiers, only 35% of lists were completed successfully. Because AlexNet is designed to build up identifiable features constructively, with discrete features becoming increasingly distinct at higher levels of the network, this demonstrates that the photographs have more “features” (defined as statistical regularities that can be identified using convolutional networks) than do the painterly stimuli. Furthermore, because only the low levels of the network allow classification to improve, it follows that the consistent traits that distinguish painterly categories are low-level and gestalt in nature (for more details, see Tanner et al. [58]).

While DLNs are a compelling hypothetical model of neurophysiological systems [59], the fact remains that AlexNet was able to classify both types of images much better than either humans or monkeys. This suggests that DLNs may not be ideally suited for modeling behavior. Experimental performance may have differed from DLN performance for any number of reasons. Although the monkeys displayed stable performance over time, human participants had only a single session in which to learn. As such, they may have performed better had they received more extensive training. On the other hand, DLN performance was not studied dynamically (with AlexNet trying to refine its classifier using task feedback). It, too, might have performed better had it been responsive to feedback.

At a minimum, AlexNet’s performance suggests that no associative model (such as the BoF algorithm) could account for these results. AlexNet’s convolutional layers collectively contain thousands of “nodes” (analogous to neurons), which are connected to one another by tens of millions of weighted connections (analogous to synapses). A network of this complexity is *necessarily* representational, in that acts as a predictive model based on past experience that can both discern and generate relevant images upon request [60]. Because DLN algorithms are a computational implementation of a representational system, these algorithms can be seen as properly cognitive, and are not recognizable as associative models.

Modern computational models force theorists to consider the distinction between the information a neuron can encode vs. the information a network can encode. The individual nodes of AlexNet may, in some sense, be considered “associative” (since they update via simple learning rules) but this does not make AlexNet collectively an associative model, any more than a spatial map encoded in the hippocampus is “associative” because its computations are performed by neurons. Given that robust representation is possible using feed-forward DLNs like AlexNet, both traditional cognitive theories (with their vague box-and-arrow diagrams) and traditional associative theories (with their simple stimulus-response relationships) will need to adapt, and to be more specific about the level of analysis at which they describe mental and behavioral phenomena.

The similarity between monkey and human performance in the case of the paintings distinguishes our stimuli from sets of man-made stimuli used in other primate studies [14, 61] which incorporated consistent features, like wheels or windows. Although paintings have been used as discriminative stimuli in animals studies by Watanabe and colleagues (e.g. [10, 62, 63]), ours is the first study to use painting stimuli with non-human primates. This study also presented subjects with a much more difficult task than previous studies. Subjects had to classify stimuli from four different categories simultaneously. In the painting condition, these exemplars used only small segments of the full canvas. The difficulty of the task and the ambiguity of the stimuli both helped to ensure that categorization performance could not be ascribed to simple associative mechanisms. Most studies of animal categorization require that subjects discriminate between only two categories, and such tasks can be solved using shortcuts that would fail if more than two categories were presented simultaneously [40].

### Implications and conclusions

The ability to categorize percepts has been found in multiple species of primate [4, 6, 9], as well as pigeons [5, 8, 63]. Accordingly, our results are consistent with our finding that non-human primates can flexibly classify stimuli according to abstract stimulus properties. Additionally, both humans and macaques have previously been shown to attend to the overall family resemblance of sets of stimuli, even in cases when doing so impairs performance because the task requires selecting on the basis of only a single feature [34].

Our study suggests other avenues for future work that could further develop this result. We explicitly provided as little information as possible to our human participants in advance of the experimental task. For example, if human participants were trained in art history, then having a formal framework should permit linguistic classification, with a corresponding improvement in performance. None of our participants were art majors, so it might also be revealing to compare trained artists or art historians to amateurs, to see if formal training would yield performance more in line with the rapid learning displayed with the photographic stimuli.

More research is needed to understand the specific operations of the classifiers that monkey use to categorize stimuli. Future investigations will benefit from more demanding experimental designs that use larger stimulus sets, more categories, and harder tasks. The present result suggests that serial lists of abstract categories can be manipulated in the same fashion as fixed stimuli. A way to corroborate this aptitude would be to use categories with changing stimuli to train a transitive inference task, which appears to tap into the same representational mechanisms as SimChain [43].

Understanding complex learning in animals has always been difficult because animals cannot learn human languages. Our results suggest that language makes internal percepts significantly easier to classify, but we have also shown that stimuli can be integrated into percepts without the benefit of language. An animal’s lack of human language is most often seen as an obstacle to complex learning, but out results show that non-linguistic animals can teach us much about the structure of cognition.

## Supporting information

S1 File Supplemental information. Details of stimuli, descriptive stimuli analyses, and human model learning parameters.
(PDF)

S2 File Data and analyses. All data and all analytic code used in this manuscript.
(ZIP)
