# Supplementary material for: Perceptual category learning of photographic and painterly stimuli in rhesus macaques (Macaca mulatta) and humans
Source: PLoS One. 2017 Sep 29;12(9):e0185576. doi: 10.1371/journal.pone.0185576 (PMC5621688; doi:10.1371/journal.pone.0185576)
Supplement: S1 File — Details of stimuli, descriptive stimuli analyses, and human model learning parameters. (PDF) [file pone.0185576.s001.pdf]

Perceptual Category Learning of Photographic and Painterly Stimuli in Rhesus Macaques (*Macaca mulatta*) and Humans: Supplemental Information

Drew Altschul[1,2], Greg Jensen[3], and Herbert Terrace[3]
[1]The University of Edinburgh
[2]Scottish Primate Research Group
[3]Columbia University

Stimuli

Although comparative studies of primate cognition very often use photographic stimuli, systematic analyses of the stimuli are rarely undertaken. This is unfortunate, because skepticism about surprising results often relies on speculation about stimulus characteristics that *might* have been used as discriminative cues. A subject might, for example, identify pictures of birds solely on the basis of blue backdrops (i.e. the sky). Analysis can determine whether bird stimuli contain disproportionate amounts of blue.

Our analysis of stimulus images focuses on low-level features (e.g. color, image entropy). This approach has the advantage of being automatic and replicable. The overarching question that these analyses seek to inform is this: "To what extent are low-level properties sufficient to categorize stimuli correctly?" More diverse stimuli in each grouping means more difficulty specifying criteria for category inclusion. At the same time, the more each of the categories resembles the others overall, the more difficult it is to specify criteria for category exclusion.

The stimuli used in this study were selected in a fashion that differs from the conventions used in typical psychophysical experiments. Rather than select stimuli according to strict inclusion criteria, or modifying images before use (e.g., turning them grayscale or giving them uniform spectra), we included images solely on the basis of the question, "Is this a picture of X?" For example, our photographs of people included both extreme close-ups of faces and wide-angle views of crowds. In cases of other animals, some part of the head was usually visible, but not necessarily from any particular angle, and the photos included pictures of packs or flocks of animals in some cases. Additionally, we also included both color and black-and-white images.

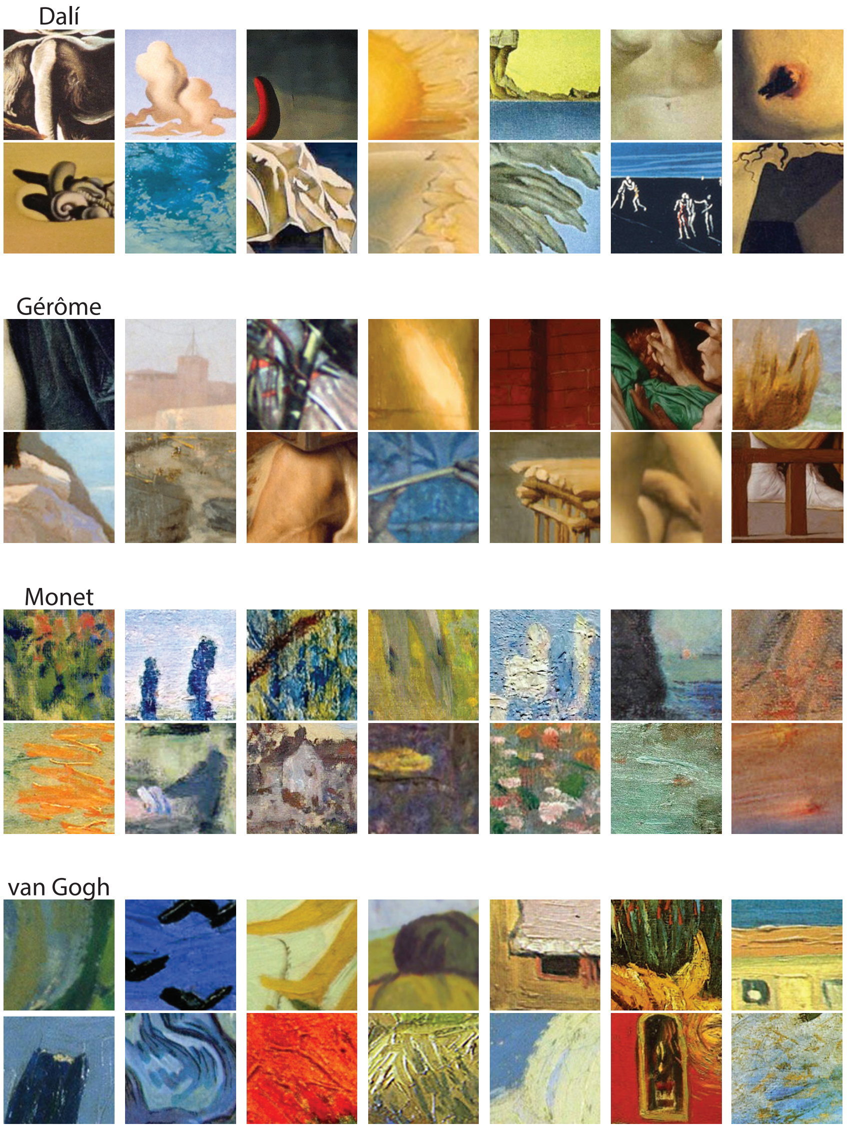

Figure 1: Exemplars of the stimuli drawn from the works of four painters, used with monkeys and humans.

The photographic stimulus sets were birds (3111 images), cats (1114 images), flowers (3033 images), and people (2853 images). The painting stimulus sets were derived from works by Dalí (400 images), Gérôme (406 images), Monet (400 images), and van Gogh (400 images).

Figures 1 depict a representative set of exemplars for each of the painting categories. For reasons associated with image and likeness rights, exemplars from the photographic categories are not included. However, representative images may be obtained from the Caltech-UCSD Birds 200 Dataset (Welinder et al., 2010) for the 'birds' category, from the Oxford-IIIT Pet Dataset (Parkhi et al., 2012) for the 'cats' category, from the Oxford 102 Category Flower Dataset (Nilsback and Zisserman, 2008) for the 'flowers' category, and from the Caltech 256 Dataset (Griffin et al., 2007) for the 'people' category. Subjects with prior experience using the SimChain paradigm were not previously exposed to any of the images in each of the categorical image banks.

Stimulus Analysis: Pixel Entropy

Prior research has shown that primates possess the ability to discriminate stimuli based on visual entropy Flemming et al. (2013); Wasserman et al. (2001), an ability also demonstrated in pigeons Young and Wasserman (2002). Because the entropy estimation can be done mechanically by simple systems, doing so falls considerably short of the criteria for a "categorical representation." Consequently, an analysis of pixel entropies gives an idea of whether the sets of stimuli differ sufficiently to be discriminated on that basis.

Here, pixel entropy is taken to be the Shannon entropy Jensen et al. (2013), computed over all possible combinations of red, blue, and green intensities:

$$H = \sum_{r=0}^{255} \sum_{g=0}^{255} \sum_{b=0}^{255} p\left(r, g, b\right) \dot{\log}_2 \left(p\left(r, g, b\right)\right) \quad (1)$$

The maximum possible entropy H that a bitmap image could possibly display is 24, provided each of the $256 \times 256 \times 256$ pixel values appears equally. However, such an entropy would require a 4096×4096 pixel image, much larger than our stimuli. Because our stimuli were only 200×200 pixels in size, the highest possible entropy that a color stimulus could possess was 15.29 bits. Grayscale images had a maximum entropy of 8 bits.

Figures 2 and 3 show kernel density estimates of the distributions of pixel entropies displayed in the photographic and painterly categories, respectively, as well as each distribution's quartiles. In general, stimuli tended to show high entropies of between 12 and 14 bits, such that a 13-bit image could easily belong to any of the categories. However, the photographic stimuli show clear distributional differences. For example, many more of the images of birds have entropies below twelve than the other stimuli, while the images of flowers routinely have higher entropies than the other stimuli.

The painting stimuli tend to have higher entropies overall than the photographic stimuli. Here, too, however, there are notable similarities. Dalí and

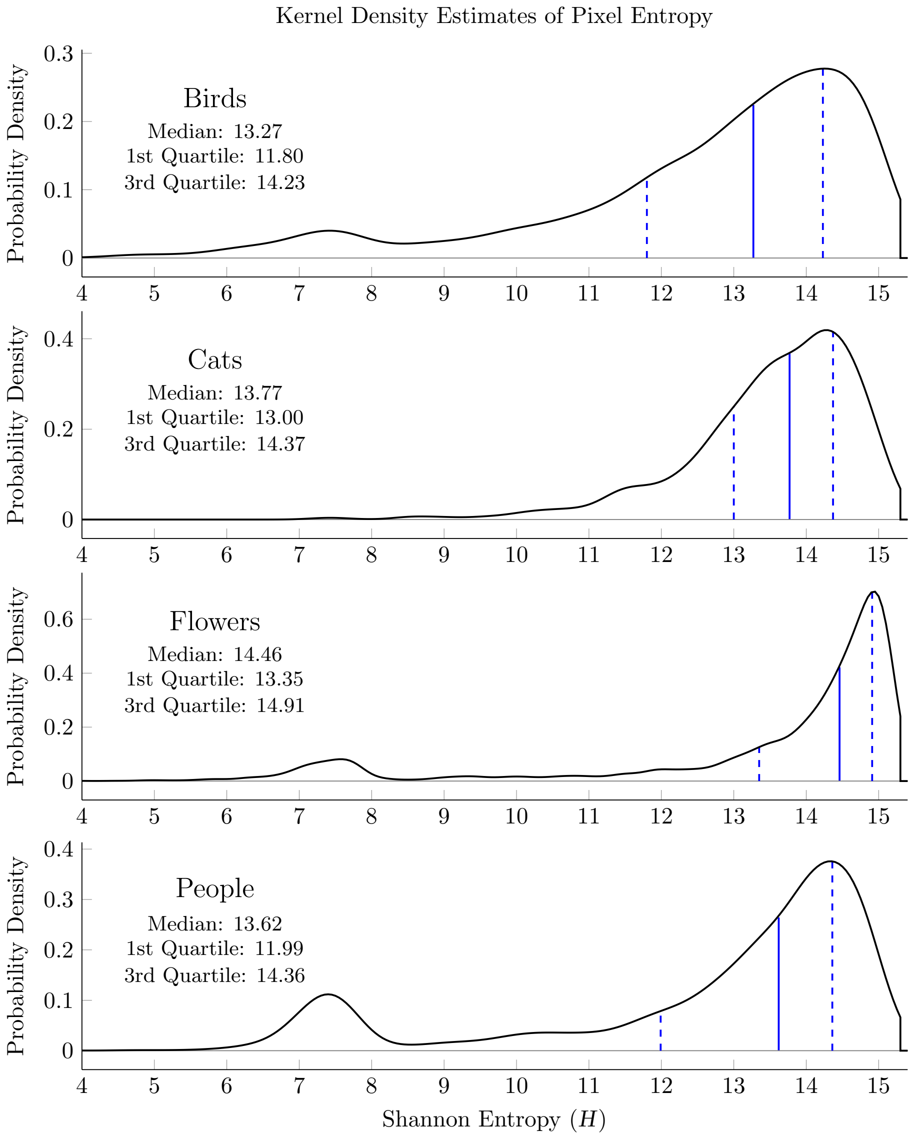

Figure 2: Kernel density estimates of pixel entropy in the four photographic categories. The median image is indicated by the solid blue line, while the first and third quartiles are indicated by the blue dashed lines.

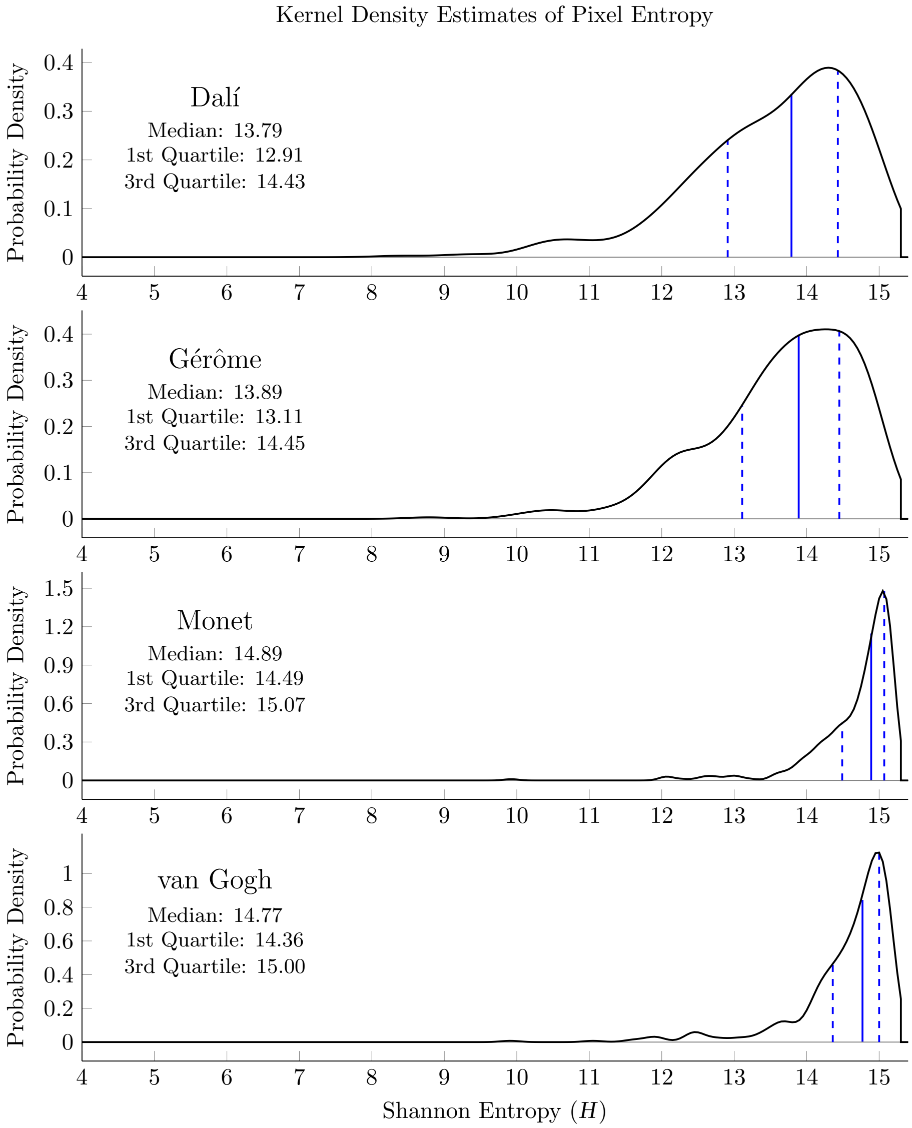

Figure 3: Kernel density estimates of pixel entropy in the four photographic categories. The median image is indicated by the solid blue line, while the first and third quartiles are indicated by the blue dashed lines.

Gérôme both resemble one another closely, as do Monet and van Gogh, but these two clusters appear distinct from one another. Importantly, however, because stimuli in each of these pairings are distributed so similarly, it would be very difficult for subjects to distinguish each group precisely on the basis of pixel entropy alone.

We do not rule out the possibility that pixel entropy facilitated identification in some fashion. This analysis is merely intended to demonstrate that pixel entropy alone would not have been sufficient to precisely classify each stimulus.

Stimulus Analysis: HSV Histograms

Another method by which images can be compared is on their HSV distributions. Just as each image may be represented as a collection of pixels that have values of red, blue, and green, each pixel may also be represented by the orthogonal dimensions of hue, saturation, and value (the last corresponding to the luminosity of the pixel). HSV histograms are often more subjectively informative than RGB histograms, as they are better at revealing effects such as tint, brightness, and color intensity Lee et al. (2005).

For this analysis, the histograms of hue, saturation, and value were obtained for each stimulus. Then, these stimuli were sorted the position of the peak in each distribution. This yields a 3D map of frequencies across stimuli, in which each row represents a single stimulus and each column represents a particular index in the histogram.

Figure 4 plots this multi-image histogram as a heat map for the hues of all photographic stimuli. In addition to the histograms for each individual image, Figure 4 also plots the marginal frequencies across *all* stimuli in each category. Here, we can see quite clearly that the different categories reliably have properties that can be used to distinguish one category from the next. Pictures of birds very frequently have green and cyan elements (because of leaves or sky), and flowers have a greater representation of yellow and purple. Photographs of people tend to be more reddish, while cats tend to be more orange.

Note that the apparently "blank bands" visible in these heat maps are black-and-white images. Since a black-and-white image cannot reasonable be described as having a particular hue, the frequency distribution for those images were uniform.

Figure 5 plots the histogram for saturation of photographic stimuli, and here, too, patterns of differ visibly. Photographs of flowers are typically highly saturated, while photos of cats and birds tend to have low saturation. However, an examination of the distributions of individual stimuli suggest that there is an overall level of heterogeny in most cases, as evidenced by the lack of consistent vertical bands in the heat maps.

Figure 6 plots the histogram for value (i.e. brightness) of photographic stimuli, showing clear differences. Flowers and people tend to be spread across the range, while birds and cats tend to cluster toward the center. As in the case

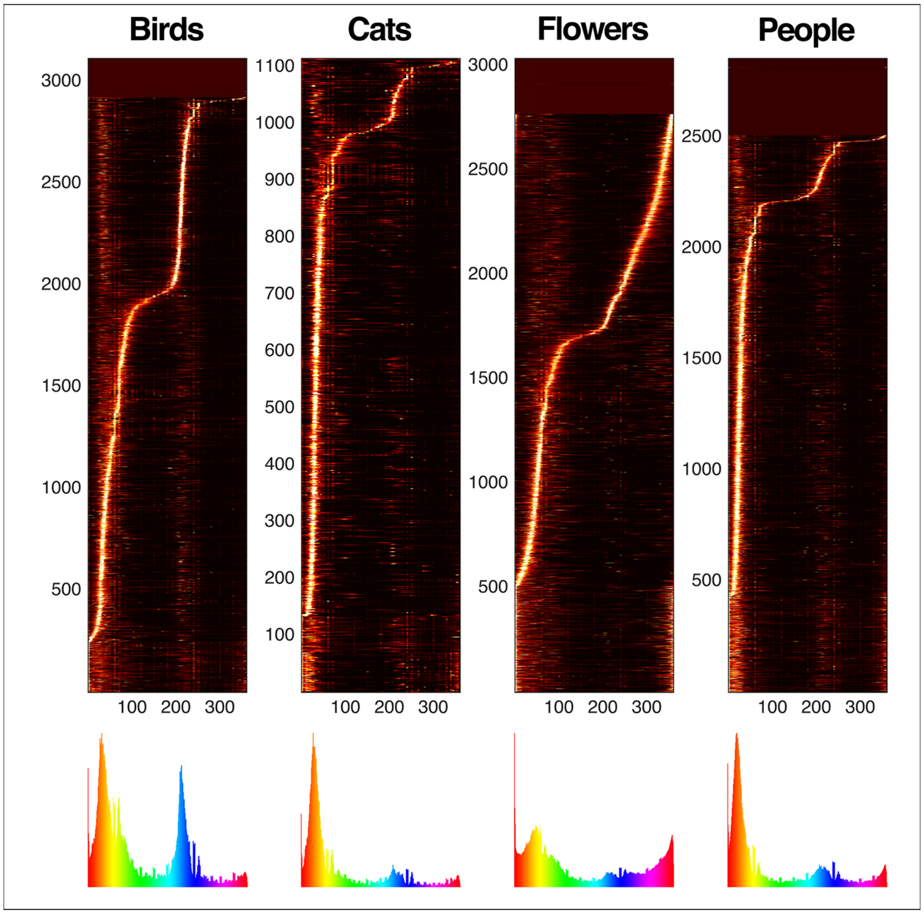

Figure 4: Hue histograms for each photographic stimulus category, sorted by peak frequency. The histogram at the bottom depicts the marginal frequency across all images.

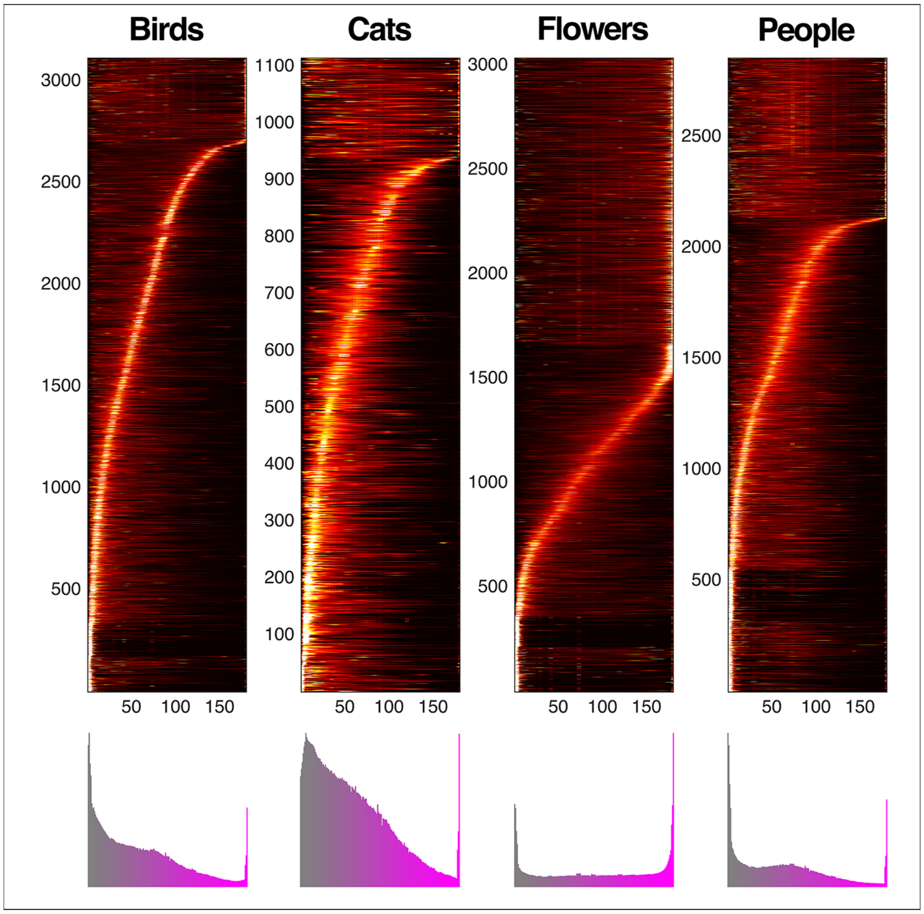

Figure 5: Saturation histograms for each photographic stimulus category, sorted by peak frequency. The histogram at the bottom depicts the marginal frequency across all images.

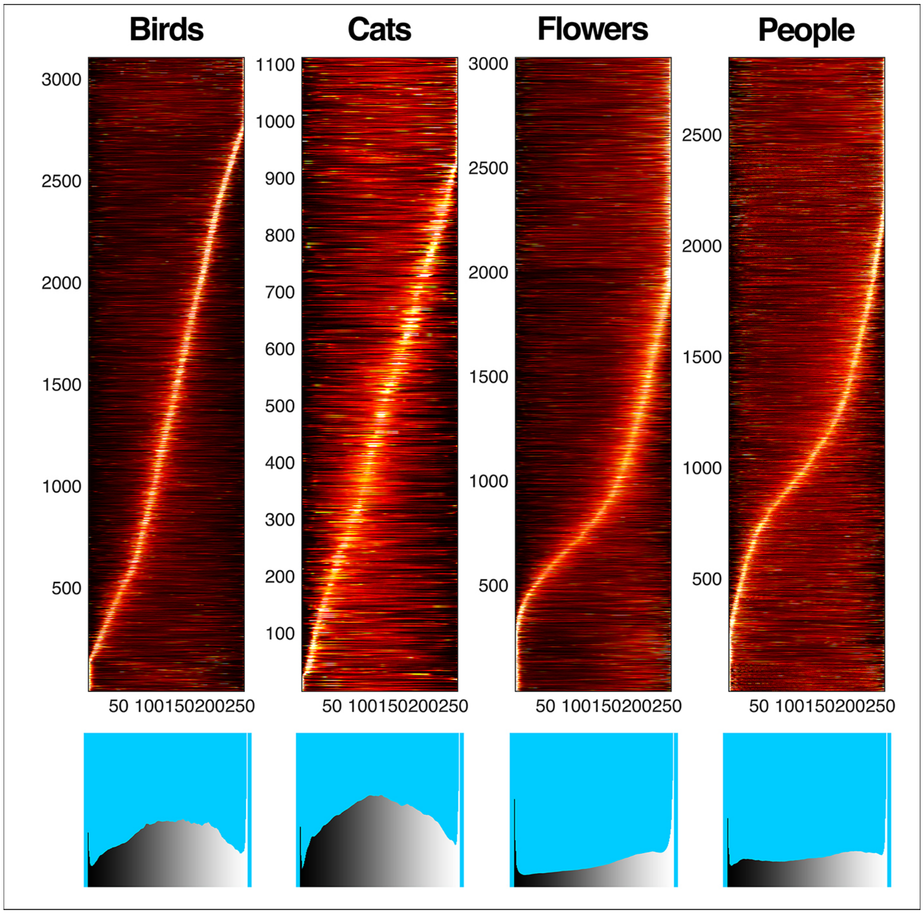

Figure 6: Value histograms for each photographic stimulus category, sorted by peak frequency. The histogram at the bottom depicts the marginal frequency across all images.

of saturation, there is a great deal of variation across stimuli, such that these would not be strongly selective signals.

Figure 7 plots the histogram for hue of the painting stimuli, and a much greater degree of uniformity is observed here than in the photographic stimuli. In all cases, painters favored colors in the orange-yellow and cyan ranges. This similarity across painters, combined with the heterogeny of the images (in which many had no blue to them at all) ensures that hue could not be used as a reliable cue for differentiating between painters.

Figure 8 plots the histogram for saturation of the painting stimuli. Here, heterogeny dominated, with all painters having images that were spread across the full range of saturations. While there were some differences (Dalí tended to be the most likely to have highly saturated colors, for example), the spread across the range prevented saturation from being a reliable cue.

Figure 9 plots the histogram for value of the painting stimuli. As in the case of saturation, the painters were highly heterogeneous, tending to favor intermediary values. This is unsurprising, as artists routinely avoid using pure white and pure black, instead favoring intermediate values that give an impression of contrast Escher (1989).

Human Learning Model Parameters

Parameter estimation for SimChain learning is complicated by two features of the model. The first (a problem in all non-linear regression regimes) is that the four learning parameters covary with one another. For example, if the twist parameter v is doubled, this has the effect of shifting the entire distribution to the left, forcing the peak parameter m to reduce its value accordingly.

The second problem is that when performance is close to either the floor or the ceiling, it becomes impossible to differentiate between values for the other parameters. For example, if the learning rate is close to zero, then the twist and peak parameters could have an enormous range of values without substantively impacting the shape of the learning curve.

Although Stan (Carpenter et al., 2015) is capable of robust inference regarding multi-level non-linear models, it cannot provide precise information about undecidable parameter values. This can be seen in Figure 10, which displays the learning parameters using photographic categories (top row) and painting categories (bottom row). In both cases, the peak parameter m and the twist parameter v showed poor convergence, as a consequence of both parameter covariance and parameter undecidability when learning rates were low.

A clearer picture emerged in the case of a model of the learning rates, depicted in Figure 11, which showed much more stable convergence. In general, reaction times were initially slower for early list items than for late list items, as shown by the downward trend in the intercept parameters k. Participants also tended to respond more rapidly as time passed, as evidenced by distributions of slope parameters b tending to be less than zero.

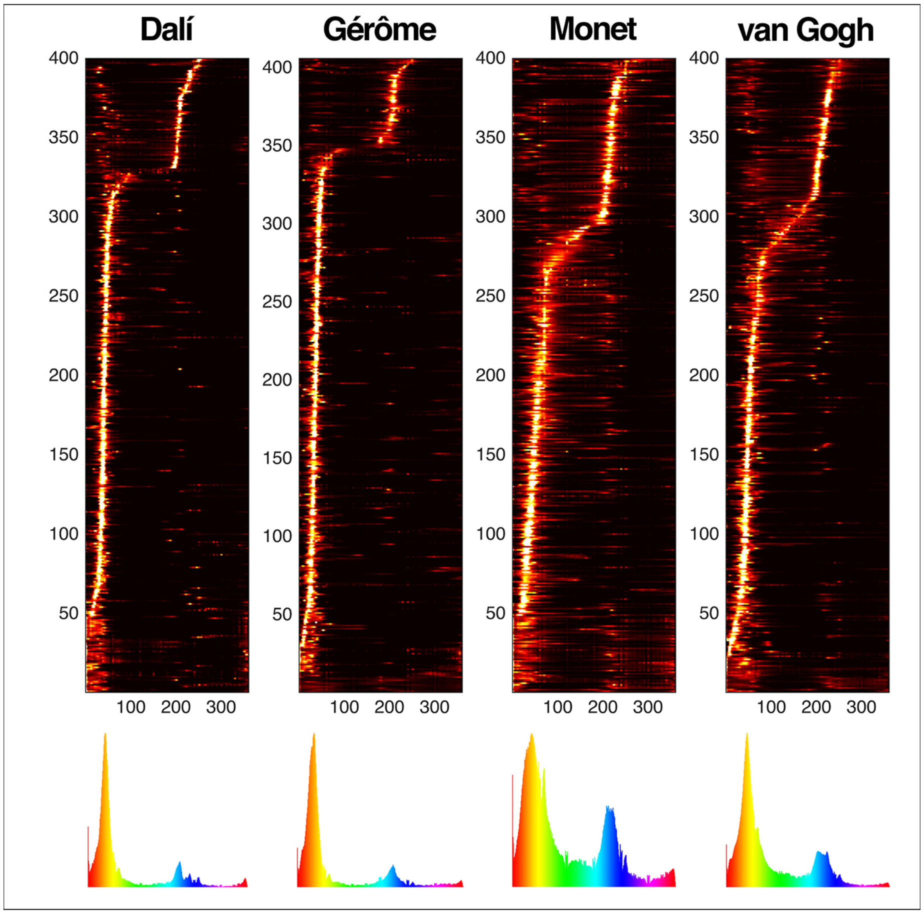

Figure 7: Hue histograms for each painterly stimulus category, sorted by peak frequency. The histogram at the bottom depicts the marginal frequency across all images.

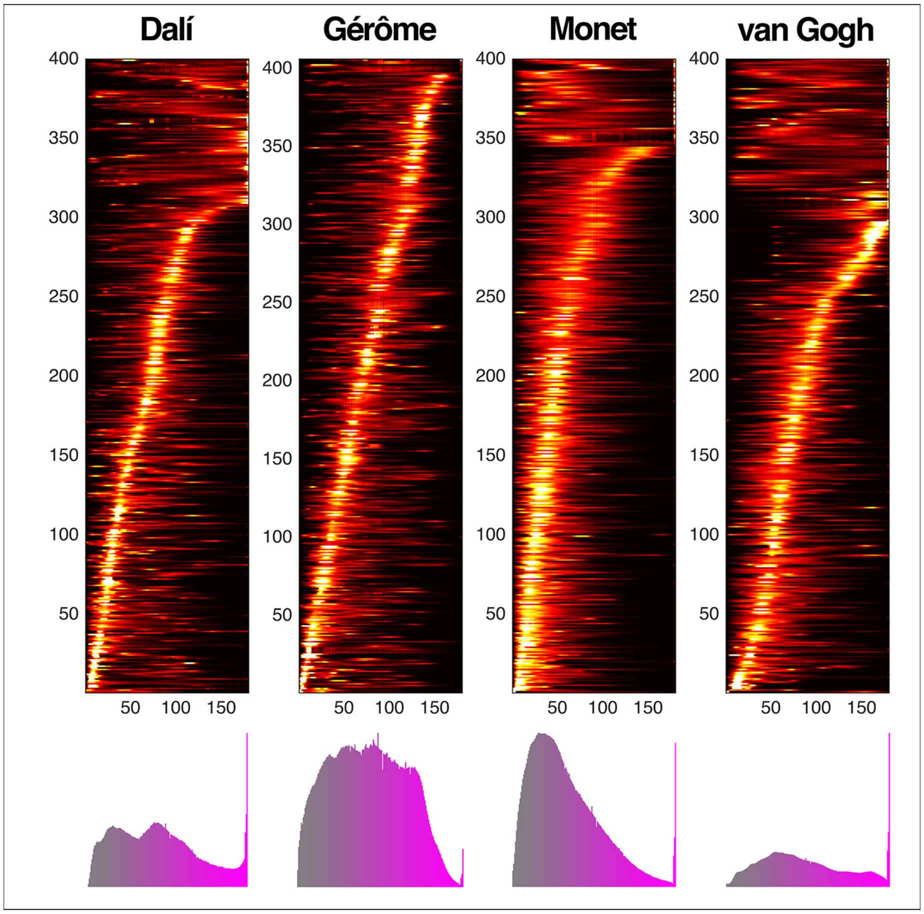

Figure 8: Saturation histograms for each painterly stimulus category, sorted by peak frequency. The histogram at the bottom depicts the marginal frequency across all images.

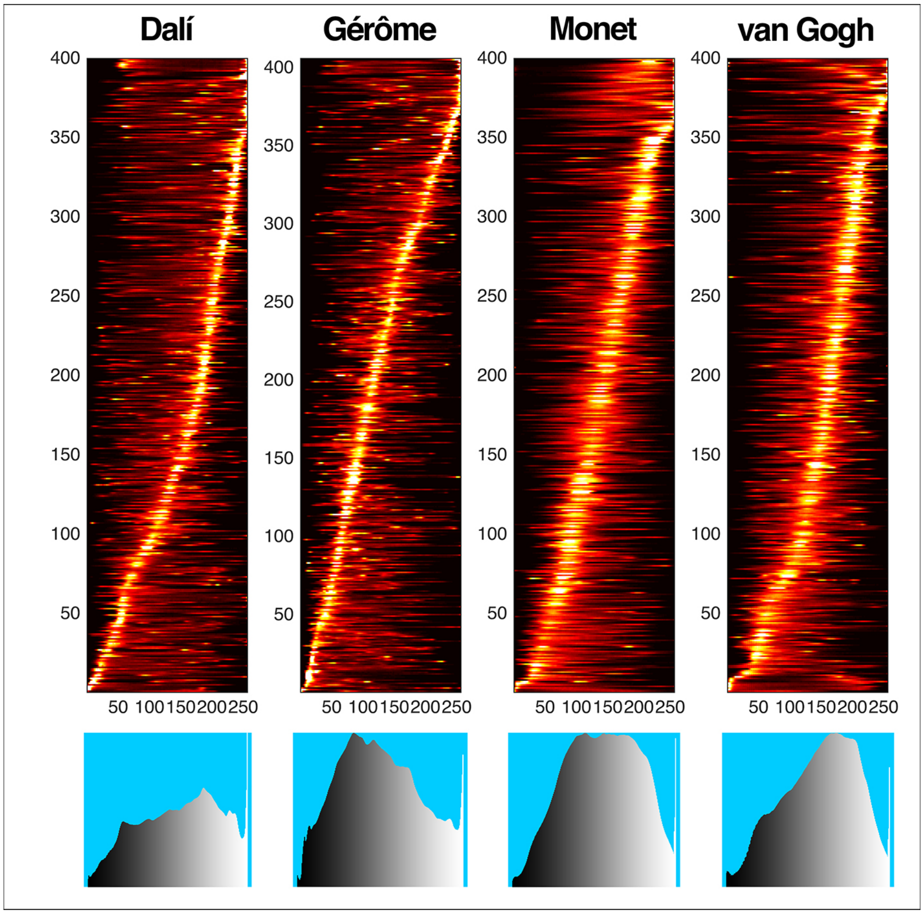

Figure 9: Value histograms for each painterly stimulus category, sorted by peak frequency. The histogram at the bottom depicts the marginal frequency across all images.

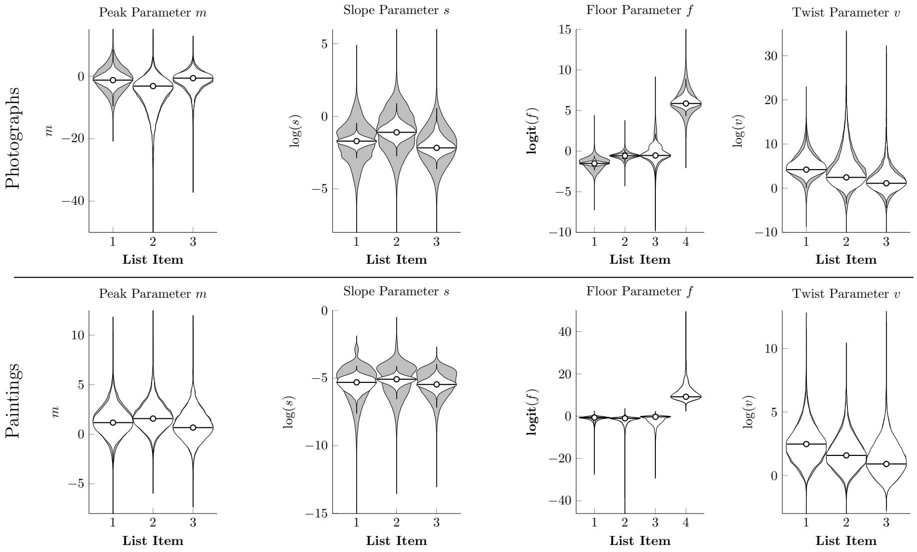

Figure 10: Estimated population distributions for human performance on the Category Chain. Gray violin plots represent the posterior population distribution for each parameter, whereas white violin plots represent the credible interval for the mean of those distributions. **Top Row**. Learning rate parameters during phase 1, using photographic stimuli. **Bottom Row**. Learning rate parameters during phase 2, using painting stimuli.

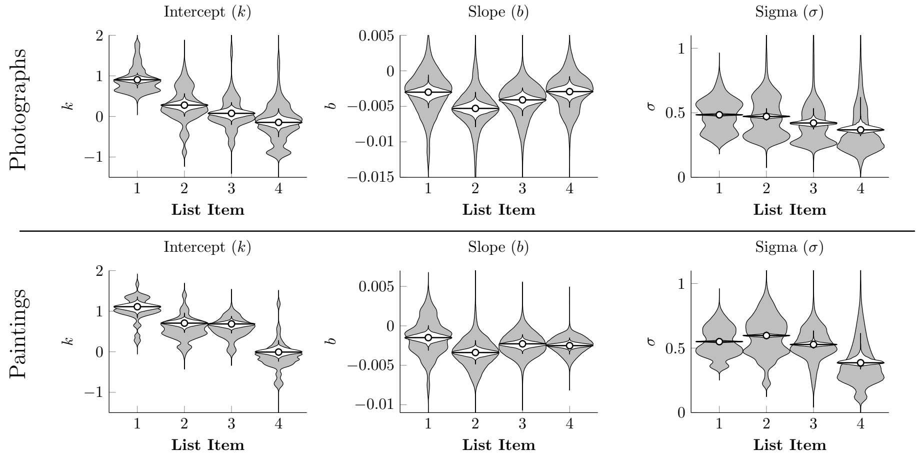

Figure 11: Estimated population distributions for linear models of reaction times on the Category Chain. Gray violin plots represent the posterior population distribution for each parameter, whereas white violin plots represent the credible interval for the mean of those distributions. **Top Row**. Reaction time model for phase 1, using photographic stimuli. **Bottom Row**. Reaction time model for phase 2, using painting stimuli.

References

Carpenter, B., Gelman, A., Hoffman, M., Lee, D., Goodrich, B., Betancourt, M., Brubaker, M. A., Guo, J., Li, P., and Riddell, A. (2015). Stan: A probabilistic programming language. *Journal of Statistical Software*.

Escher, M. C. (1989). *Escher on Escher: Exploring the Infinite*. Harry N. Adams, New York, NY.

Flemming, T. M., Thompson, R. K., and Fagot, J. (2013). Baboons, like humans, solve analogy by categorical abstraction of relations. *Animal Cognition*, 16:519–524.

Griffin, G., Holub, A. D., and Perona, P. (2007). The Caltech 256. Technical Report CNS-TR-2007-001, California Institute of Technology.

Jensen, G., Ward, R. D., and Balsam, P. D. (2013). Information: Theory, brain, and behavior. *Journal of the Experimental Analysis of Behavior*, 100:408–431.

Lee, S. M., Xin, J. H., and Westland, S. (2005). Evaluation of image similarity by histogram intersection. *Color Research and Application*, 30:265–274.

Nilsback, M.-E. and Zisserman, A. (2008). Automated flower classification over a

large number of classes. In *Proceedings of the Indian Conference on Computer Vision, Graphics and Image Processing*.

Parkhi, O. M., Vedaldi, A., Zisserman, A., and Jawahar, C. V. (2012). Cats and dogs. In *IEEE Conference on Computer Vision and Pattern Recognition*.

Wasserman, E. A., Fagot, J., and Young, M. E. (2001). Same-different conceptualization by baboons (*Papio papio*): The role of entropy. *Journal of Comparative Psychology*, 115:42–52.

Welinder, P., Branson, S., Mita, T., Wah, C., Schroff, F., Belongie, S., and Perona, P. (2010). Caltech-UCSD Birds 200. Technical Report CNS-TR-2010-001, California Institute of Technology.

Young, M. E. and Wasserman, E. A. (2002). The pigeon's discrimination of visual entropy: A logarithmic function. *Animal Learning and Behavior*, 30:306–314.
